# Characteristics and Risk Assessment of Soil Polluted by Lead around Various Metal Mines in China

**DOI:** 10.3390/ijerph18094598

**Published:** 2021-04-26

**Authors:** Jing Shi, Ping Du, Huilong Luo, Juan Chen, Yunhui Zhang, Minghong Wu, Gang Xu

**Affiliations:** 1 School of Environmental and Chemical Engineering, Shanghai University, Shanghai 200444, China; sj960314skinner@outlook.com (J.S.); mhwu@shu.edu.cn (M.W.); xugang@shu.edu.cn (G.X.); 2 Technical Centre for Soil, Agriculture and Rural Ecology and Environment, Ministry of Ecology and Environment, Beijing 100012, China; huilong.luo@mail.bnu.edu.cn (H.L.); chenjuan@craes.org.cn (J.C.); lchjj2012@163.com (Y.Z.); 3 College of Water Science, Beijing Normal University, Beijing 100875, China

**Keywords:** blood lead level, ecological risk, metal mine, pollution characteristics, soil lead

## Abstract

The contamination of soil by lead (Pb) is a serious and widespread problem in China, especially in mining areas. This paper summarized the available data regarding Pb-contaminated soils around various metal mines in China. Based on these data, the Pb concentration in the soil and its temporal and spatial changes were analyzed. Potential ecological hazards and adult lead models were also used to estimate ecological and health risks. The results indicated that the concentration of Pb was closely related with the type of mine. Compared with other types of mine, soil around lead-zinc (Pb-Zn) and tin (Sn) mines with high Pb contents in the metallic ores and high pollutant emission coefficient were more strongly polluted by Pb. The characteristic spatial and temporal variations of Pb pollution status in China were clarified, and the results showed that the concentration was high in the southern, southwestern, and central regions of China where many mining areas were located, and the mean value passed a turning point in 2012. Ecological risk assessments indicated that some areas around mines were at considerable to high risk, and the risk was relatively severe in Pb-Zn mining areas. According to the adult lead model, Pb-Zn mines had a greater impact on blood Pb concentration than the other types of mine.

## 1. Introduction

Lead (Pb) is considered one of the most toxic trace elements in the environment, and it potentially can cause serious health problems in humans, animals, and plants [1,2,3,4]. Many studies have investigated the pollution of soil with Pb, including environmental and health issues across China. According to the first National Soil Pollution Investigation of China, 1.5% of all soil samples were contaminated with Pb [5] and mining activities were considered one of the most significant sources of heavy metal contamination among human industrial, mining, and farming activities. According to statistics, China is rich in mineral resources, and Pb reserves and output rank among the top in the world [6]. Heavy metals may be released into the surrounding environment during the mining process and finally accumulate in soil, thereby resulting in high soil Pb concentrations around mining areas [7,8]. Learning from most studies of the pollution of soils with heavy metals related to mining activities in recent years, Pb-Zn mines are considered to have a serious impact on soil Pb pollution [9,10,11]. In addition, risk assessments have been conducted regarding the pollution of soil with heavy metals from mines. Li et al. [12] performed pollution and health risk assessments for soils in 72 mining areas and proposed that Pb, cadmium, arsenic, and other pollutants should be prioritized for control. These conclusions are very important for determining the key elements that need to be controlled during environment management.

Most of the studies mentioned above focused on only a single or several mining areas, whereas few have considered the different contributions and impacts of multiple mines. Thus, a comprehensive pollution assessment is urgently needed on a national scale for mining areas in China, especially determining the differences in the potential risk associated with various mines. The aims of this research were: (1) to evaluate the soil Pb pollution around various metal mines in China; (2) to analyze the ecological risks and blood lead levels (BLLs) associated with different types of mines; and (3) to propose policy recommendations for relevant agencies. The conclusions obtained in this study could be useful for developing management policies and control measures for different mines.

## 2. Materials and Methods

### 2.1. Data Collection and Analysis

Using the search terms “Pb, soil, mine, China”, 154 papers (Table S1) published between 2002 and 2016 were collected from the Web of Science, Elsevier Science Direct, Chinese Periodical Full-text, China National Knowledge Infrastructure (CNKI), Science Online, and other sources. The samples were all surface soil samples (depths of 0–15 cm or 0–20 cm) obtained using conventional sampling methods, such as snake-type sampling, gridding sampling, multipoint sampling, and random sampling. The samples were mainly analyzed by inductively coupled plasma-mass spectrometry, graphite furnace atomic absorption spectrophotometry, or atomic absorption spectroscopy. The average values recorded in previous studies were used to represent the concentrations at the sampling sites (Table S1).

**Figure 1:**
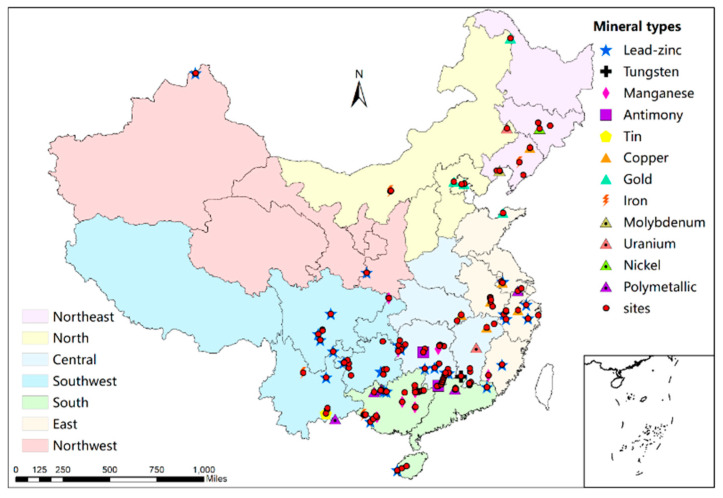
Distributions of sites and different mining areas in China.

In total, 258 sites with Pb contaminated soils were identified in studies of mining areas distributed across 21 provinces throughout China (Figure 1). Each study investigated only one or a few sites with suspected Pb contamination. Based on these studies, we conducted a relatively comprehensive, national-scale assessment of Pb contamination in soils around typical metal mines in China.

### 2.2. Ecological Risk Assessment

The ecological risk index developed by Hakanson has been used widely to evaluate the potential ecological hazard due to heavy metals [13,14,15]. This method combines environmental chemistry, biotoxicology, and ecology to assess the effects of various pollutants and the comprehensive effects of various pollutants. The ecological risk index is calculated as follows:

$$E_{f}^{i}=T_{i}\times \frac{C_{i}}{C_{0}}\;RI=\overset{n}{\underset{i=0}{\sum }}E_{f}^{i}$$

where $E_{f}^{i}$ and *RI* are the single ecological risk index and multiple ecological risk index, respectively, *C_i_* is the concentration of Pb measured in soil (mg/kg), *C*_0_ is the reference value, i.e., the screening value for the soil environmental quality standard (GB 15618–2018), and *T_i_* is the toxicity response coefficient. The toxicity response coefficient for Pb is 5 based on Hakanson’s standardized toxicity response coefficient. The risk classes based on $E_{f}^{i}$ can be classified into five levels (Table 1; Wang et al. [16]).

### 2.3. Adult Lead Model (ALM)

The ALM was developed to estimate the blood lead level (BLL) from non-contact intensive indoor occupational exposure to contaminated soil at Superfund sites in the United States [17]. The residents near mining areas could experience significant and chronic exposure to contaminated soil, which we assumed would be similar to the exposure scenario for the ALM. The ALM uses a simplified biokinetic model to predict the quasi-steady state BLL for adults under chronic exposure to lead-contaminated soil [18]. It involves the process of soil ingestion, absorption after ingestion, and conversion into blood lead after absorption. The basic formula [19] is as follows:

$$PbB_{adult,central}=PbB_{adult,0}+\frac{PbS\cdot BKSF\cdot IR_{S}\cdot AF_{S}\cdot EF_{S}}{AT}$$

where *PbB_adult,central_* is the central estimate of the blood lead concentration (μg/dL) in adults, *PbB_adult_*_,0_ is the typical blood lead concentration (μg/dL) in adults, *PbS* is the soil lead concentration (μg/g), *BKSF* is the biokinetic slope factor (μg/dL blood lead increase per μg/day lead uptake), *IR_S_* is the intake rate of soil (g/day), *AF_S_* is the absolute gastrointestinal absorption fraction (unitless), *EF_S_* is the exposure frequency for contact with the assessed soil (days/year for long term exposure), and *AT* is the averaging time (days/year). Table S2 provides more information about these parameters.

### 2.4. Statistical Analysis

The normality test was conducted before making comparisons of Pb concentrations in regional distribution and temporal trend. Pb concentrations in different regions did not comply with the normal distribution. Therefore, there was no difference analysis between different regions, just a statistic. All statistical analyses were conducted using SPSS.

## 3. Results and Discussion

### 3.1. Pollution Status of Soil Pb around Mines in China

**Figure 2:**
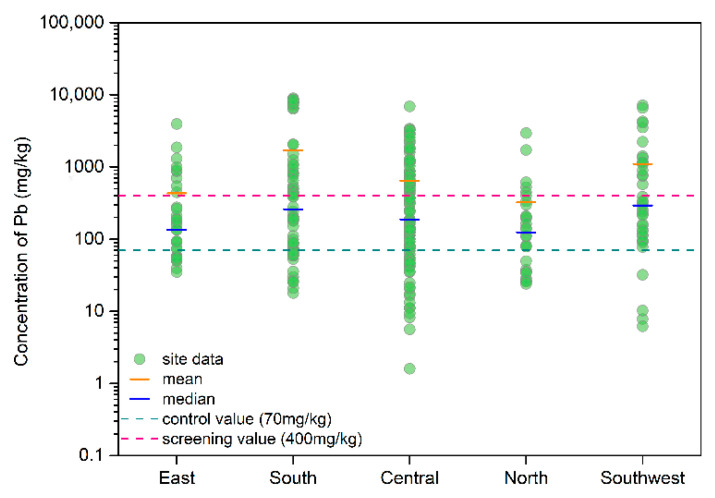
Regional distribution of Pb concentrations (East region includes Zhejiang, Anhui, Shanghai, Jiangxi, Shandong, Jiangsu and Fujian provinces. South region includes Guangxi, Guangdong and Hainan provinces. Central region includes Hunan, Hubei and Henan provinces. North region includes Shanxi, Shaanxi, Hebei, Beijing, Tianjin, Inner Mongolia, Jilin, Heilongjiang and Liaoning provinces. Southwest region includes Yunnan, Guizhou, Chongqing and Sichuan provinces).

In total, 63 major metal mines were identified in 21 provinces throughout China in this study (Figure 1). In total, 11 types of mines were identified and the main types were Pb-Zn, Sn, tungsten (W), copper (Cu), manganese (Mn), gold (Au), antimony (Sb), and iron (Fe) mines (Table S3). The sites considered were mainly located in southern and central China, but especially in Hunan and Guangxi provinces, which are rich in mineral resources and the locations of non-ferrous metal reserves [20,21]. Based on the distribution of mining areas and the geographical division of China, five regions were investigated in this review. As shown in Figure 2, the Pb concentrations varied among the five regions investigated. The median concentration in all regions was between the screening value and control value (China soil environment quality standard, GB15618-2018). The southern, southwestern, and central regions have relatively higher levels of Pb contamination than other regions, and this pattern is closely related to the distribution of mines in China.

In this study, the screening (70 mg/kg) and control (400 mg/kg) values were used as references to assess the Pb contamination in soils (China soil environment quality standard, GB15618-2018). The results showed that 76.9% of the sites exceeded the screening value and 38.5% exceeded the control value. The mean Pb concentration was 542.1 mg/kg, which was about 7.7 times larger than the screening value and 1.4 times larger than the control value. Compared with Li et al.’s study [12] on heavy metal pollution in China’s mining areas, the results in this study had lower Pb concentrations. The highest value was 102.4 times larger than the screening value, and it was reported in the Pb-Zn mining area of Sichuan Province. The mean concentrations of Pb in agricultural soil in China range from 32 to 93.89 mg/kg (37.55 mg/kg, Wei et al. [22]; 48.43 mg/kg, Yang et al., [23]; 32 mg/kg, Huang et al. [24]; 93.89 mg/kg, Yang et al. [25]). Therefore, the Pb concentrations in the soils around mines are clearly higher than those in agricultural soil, mainly due to the pollution that occurs during mining activities, such as ore excavation and transportation, smelting, refining, and tailings [26].

**Figure 3:**
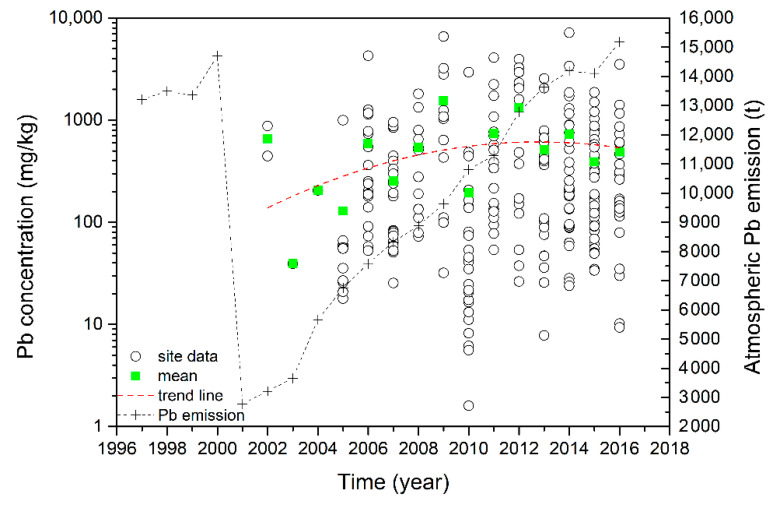
Temporal trend in Pb concentration (mg/kg) in soils in mining areas and annual atmospheric emissions of Pb (t).

Atmospheric emissions are an important source of soil Pb [27]. Generally speaking, the concentration of lead in the atmosphere near the mining area is relatively high, and atmospheric deposition as the main source has adverse effects on soil [28,29,30]. Statistical data on Pb emissions shows that atmospheric emissions showed a cliff-jumping decline in 2000 but increased gradually from 2001 to 2016. Leaded gasoline was phased out in 2000, so the atmospheric Pb emissions dropped suddenly. Subsequently, due to emissions from other sources such as coal combustion and non-ferrous smelting, the atmospheric Pb emissions slowly increased again [31,32]. In terms of temporal change, the Pb concentrations in soils around mining areas increased gradually from 2002 to 2012, and decreased slowly from 2012 to 2016 (Figure 3). This trend was consistent with that in the atmospheric emissions of Pb from 2002 to 2012, which also indicates that atmospheric Pb emissions were a significant source of soil Pb. According to the report by Shi et al. [33], Pb concentration peaked in 2006–2010, and a decrease was observed during 2011–2016, which was consistent with the results of this paper. Recently, the Chinese government adopted a series of measures to cut off the source of pollution, speed up the remediation process, and avoid further pollution, especially the 12th Five Year Plan for the control and abatement of heavy metals, which was implemented from 2011. Thus, soil Pb pollution has been controlled well in recent years.

### 3.2. Characteristics of Soil Pb Pollution around Metal Mines

**Figure 4:**
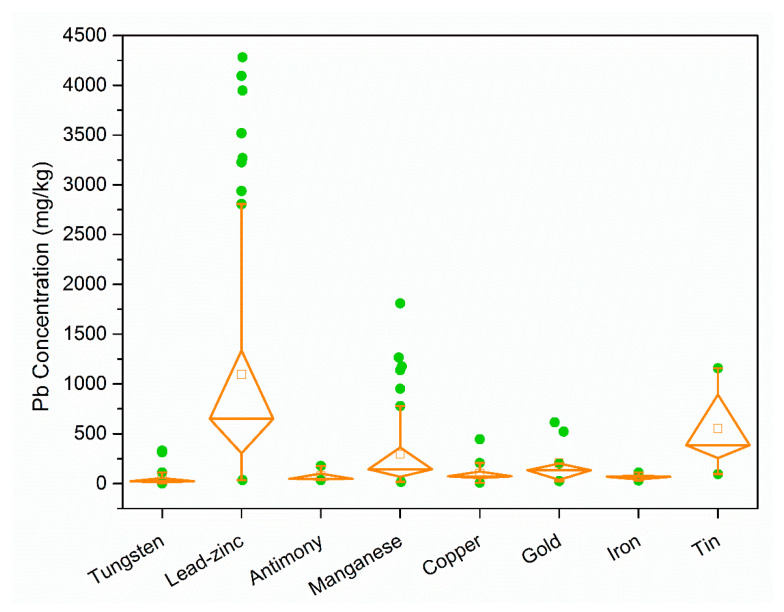
Pb concentrations in soil around different types of mines.

The soil pollution around some types of mines (i.e., around Pb-Zn and Sn mines) was relatively severe, and there was an obvious relationship between Pb pollution and different types of mines (Figure S1). The soil Pb pollution levels at eight types of metal mines are shown in Figure 4. The concentrations of Pb were relatively high around Pb-Zn and Sn mines, with average concentrations of 1095.7 and 551.43 mg/kg, respectively. In addition, the average concentrations of Pb around Au and Mn mines were about 358.8 and 294.55 mg/kg, respectively, whereas the soil Pb contents were relatively lower around Cu, Sb, Fe, and W mines.

According to Table 2, different mines have different geological characteristics and contain different associated heavy metals. Among them, Pb is the main heavy metal element in Pb-Zn mines. In addition, Pb is sometimes found in Sn, Mn, and Au mines. Analyzing the pollutant emission coefficients for industrial pollution sources showed that Pb-Zn mines had the highest Pb emission coefficient (0.262 g/t) among all of the mine types. The pollutant emission coefficient for Sn mines was 0.022 g/t, which was also much higher than that for other mine types (Table S4). It should be noted that the pollution coefficients of small and medium Sn mines were much higher than those for Pb-Zn mines, and some small-scale Sn mines had pollutant emission coefficients as high as 13.445 g/t. The annual output of Fe was 760 million tons and that of refined Cu was 9.029 million tons, but they caused far less pollution than Pb-Zn and Sn mines. The Pb emission coefficient for Cu mines was only 0.004 g/t, so the high-yield Cu mines were less polluting. In previous studies and investigations of Fe mines, Pb was not listed as the subject of investigation, thereby indicating that Pb pollution is not a concern around Fe mines. It can be seen that, without affecting the output, solving the problem of high pollutant emission coefficients in the mining process is also a good way to prevent pollution.

### 3.3. Ecological Risk Assessment

**Figure 5:**
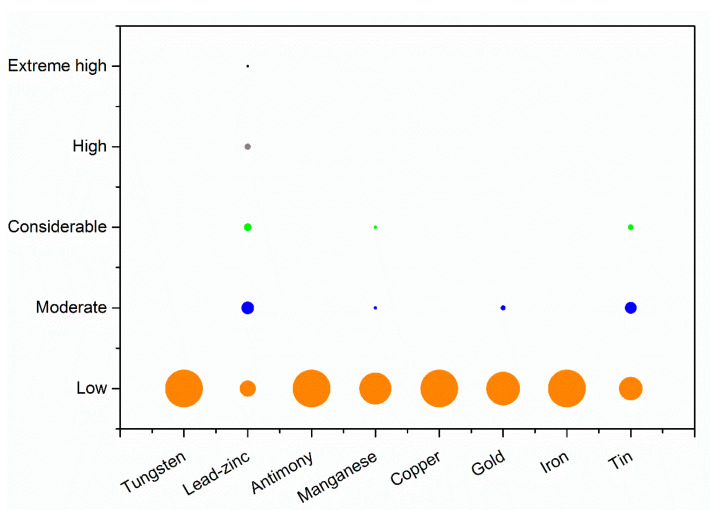
Ecological risk indexes for different types of mines.

The calculated ecological risk indexes showed that most of the sites were at low risk, where this class accounted for 72.1%, but about 13.6% of the sites were at considerable and high risk (Figure 5). The results also showed that the Pb pollution around Pb-Zn mines was mainly classified as low or moderate risk. Overall, 29.3% of the sites were at considerable to high risk. Due to the high Pb emission coefficient for Sn mines, the pollution was mainly at low to considerable risk. Other mines were basically at low risk. Therefore, greater ecological risks were detected around Pb-Zn mines and Sn mines.

### 3.4. Assessment of Adult BLLs

**Figure 6:**
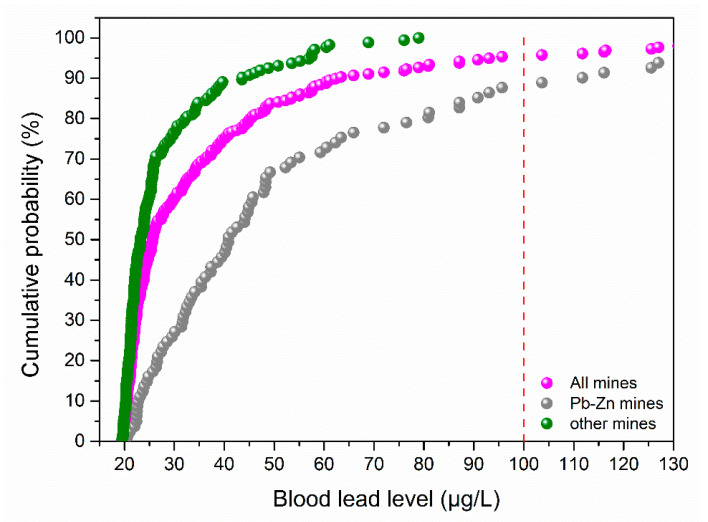
Cumulative probabilities of adult blood lead levels in mining areas.

Blood Pb poisoning is considered the biggest health problem caused by exposure to soil contaminated with Pb [34,35]. As shown in Figure 6, most of the sites were lower than the threshold value (100 μg/L) for China. In fact, about 83.72% of the sites had BLLs less than 50 μg/L and only 3.88% of the sites at Pb-Zn mines had BLLs higher than 100 μg/L. As showed in Figure 6, it can be concluded that that Pb-Zn mines had a greater impact on BLLs than the other types of mine. Only one Pb-Zn mine in Sichuan province had sites with BLLs above 100 μg/L. Thus, the soil Pb in mining areas did not pose a significant health risk to the human body.

However, it has been reported that serious cognitive impairment and neurobehavioral deficits can occur if the BLL is lower than 100 μg/L, or even 50 μg/L [36]. The threshold value for Germany (35 μg/L) and USA (50 μg/L) are both lower than that in China [37,38]. There is still no internationally recognized blood Pb poisoning threshold [39]. Thus, the higher level of 100 μg/L applied in China may underestimate the health risks caused by Pb contaminated soil. In future research, we need to pay more attention to mining areas with BLLs above 50 μg/L. Relevant departments can make further adjustments to the assessment threshold of BLL according to the actual situation in China.

## 4. Conclusions

This study reviewed Pb contamination in soils around typical metal mines in China. The results showed that the soils around mining areas are quite polluted, particularly in the southern, southwestern, and central regions of China, and the soil Pb concentrations are strongly associated with the type of mine. The pollution levels around the eight typical mine types from high to low are: Pb-Zn > Sn > Au > Mn > Cu > Sb > Fe > W mines. Temporal analysis indicated that the soil Pb is related to atmospheric Pb emissions. Without affecting the production cost and output, taking environmentally sustainable mining technologies is the first choice to reduce the Pb emission coefficient. In addition, the corresponding departments should strengthen the inspection of the process of Pb-Zn mining and the supervision of the pollution effects. Ecological risk assessment indicated that the risk is relatively severe for Pb-Zn and Sn mines. The ALM model showed that the soil around mining areas has a very limited impact on the BLL, but the impact of Pb-Zn mines is greater than those of other mines. In general, risk management for Pb-Zn mines should receive more attention in the future. It is necessary to interrupt the exposure pathway and prevent people from exposure to high-risk contaminated soil.

## Figures and Tables

**Table 1:** Hankanson’s potential ecological hazard assessment index classes.

$\mathrm{Potential}\;\mathrm{Ecological}\;\mathrm{Hazard}\;\mathrm{Coefficient}\;(E_{f}^{i})$	Potential Ecological Hazard Degree
$E_{f}^{i}$ < 40	Low risk
40 ≤ $E_{f}^{i}$ < 80	Moderate risk
80 ≤ $E_{f}^{i}$ < 160	Considerable risk
160 ≤ $E_{f}^{i}$ < 320	High risk
$E_{f}^{i}$ ≥ 320	Extreme high risk

**Table 2:** Geological characteristics of various metal mines.

Type of Mine	Major Associated Metals
Tungsten (W)	W, Sn, Mo, Bi
Lead-Zinc (Pb-Zn)	Pb, Zn, Cd, Au, Ag, Cu, Sn
Antimony (Sb)	Sb, Au, W
Manganese (Mn)	Mn, Co, Ni, Cu, Pb, Zn
Copper (Cu)	Cu, Mo, Au, Ag
Gold (Au)	Au, Ag, Cu, Zn, Pb, Pt
Iron (Fe)	Fe, Cu, Ni
Tin (Sn)	Sn, Pb, W, Bi, Cd, Ag, Cu

Mo (Molybdenum), Bi (Bismuth), Cd (Cadmium), Ag (Silver), Co (Cobalt), Ni (Nickel), Pt (Platinum).

## Data Availability

The data presented in this study are available in supplementary material.

## Supplementary Materials

The following are available online at https://www.mdpi.com/article/10.3390/ijerph18094598/s1, Figure S1: Pollution situation around different mining areas, Table S1: Basic information of soils in 154 examined areas in China, Table S2: Parameters of blood lead model in this study, Table S3: Basic information of 63 major mining areas, Table S4: Lead pollution and emission coefficients of different min-ing industries, Table S5: Data extracted from the literature, Table S6: Data analysis results (unit: mg/kg), Table S7: Data of different regions (unit: mg/kg), Table S8: Data of different years (unit: mg/kg), Table S9: Data of different mining areas (unit: mg/kg).
