# Supplementary material for: Characteristics and Risk Assessment of Soil Polluted by Lead around Various Metal Mines in China
Source: Int J Environ Res Public Health. 2021 Apr 26;18(9):4598. doi: 10.3390/ijerph18094598 (PMC8123687; doi:10.3390/ijerph18094598)
Supplement: Supplementary file 1 [file ijerph-18-04598-s001.zip › ijerph-1152887-supplementary.pdf]

Characteristics of Soil Polluted by Lead Around Various Metal Mines in China

Jing Shi [1,2], Ping Du [2,*], Huilong Luo [2,3,] Juan Chen [2], Yunhui Zhang [2], Minghong Wu [1] and Gang Xu [1]

[1] School of Environmental and Chemical Engineering, Shanghai University, Shanghai 200444, China
[2] Technical Centre for Soil, Agriculture and Rural Ecology and Environment, Ministry of Ecology and Environment, Beijing 100012, China
[3] College of Water Science, Beijing Normal University, Beijing 100875, China
* Correspondence: author: duping@craes.org.cn (P. Du)

List of Supplementary Information

1. Table S1 Basic information of soils in 154 examined areas in China.
2. Table S2 Parameters of blood lead model in this study.
3. Table S3 Basic information of 63 major mining areas.
4. Table S4 Lead pollution and emission coefficients of different mining industries.
5. Table S5 Data extracted from the literature.
6. Table S6 Data analysis results (unit: mg/kg).
7. Table S7 Data of different regions (unit: mg/kg).
8. Table S8 Data of different years (unit: mg/kg).
9. Table S9 Data of different mining areas (unit: mg/kg).
10. Figure. S1. Pollution situation around different mining areas.

Table 1. Basic information of soils in 154 examined areas in China.

	Region	Sampling method	Sampling depth (cm)	Sampling time	Mineral species	Method of laboratory analysis	Reference
A1	Gejiu, Yunnan	Snake-type sampling	0-20	2010.4	Tin	AAS	Song et al. (2012)
A2	Hunan	Gridding sampling	0-20		Antimony	AFS	Yuan et al. (2015)
A3	Gejiu, Yunnan	Gridding sampling	0-20		Tin	ICP-MS	Huang et al. (2014)
A4	Liuzhi, Guizhou	Sector sampling	0-15		Coal	AAS	Tian et al. (2013)
A5	Laibin, Guangxi	Random sampling	0-20	2010.8-2011.6	Manganese	ICP-AES	Tang et al. (2016)
A6	Yanzhou, Shandong	Multipoint sampling	0-20		Coal	AAS	Liu et al. (2004)
A7	Shaoguan, Guangdong	Sampling along the River	0-20		Polymetallic	ICP-MS	Su et al. (2014)
A8	Shaoguan, Guangdong	Multipoint sampling	0-20		Polymetallic	FAAS	Zou et al. (2008)
A9	Shaoguan, Guangdong	Multipoint sampling	0-20	2003.10	Polymetallic	ICP-MS	Zhou et al. (2004)
A10	Shaoguan, Guangdong	Random sampling	0-20		Polymetallic	ICP-OES	Xu et al. (2008)
A11	Shaoguan, Guangdong	Multipoint sampling	0-20		Polymetallic	ICP-OES	Huang et al. (2009)
A12	Anshan, Liaoning	Multipoint sampling	10-20	2009.8	Iron	AAS	Yang et al. (2011a)
A13	Inner Mongolia	Sampling vertical the River	0-20		Gold	FAAS	Hu et al. (2014a)
A14	Daye, Hubei	Snake-type sampling	0-20		Copper	GFAAS	Sun et al. (2013)
A15	Dayi,Sichuan	Random sampling	0-15		Lead-zinc	GFAAS	Lu et al. (2014)
A16	Gejiu,Yunnan	Quincunx sampling	0-20	2014.5-2014.10	Tin	ICP-MS	Mi et al. (2016)
A17	Guizhou	Multipoint sampling	0-20	2008.8	Mercury	GFAAS	Chen et al. (2010)
A18	Hengyang,Hunan	Gridding sampling	0-15	2009.7	Lead-zinc	ICP-MS	Sun et al. (2012)
A19	Dexin,Jiangxi	Multipoint sampling	0-20	2004.4	Copper	ICP	Chen et al. (2007)
A20	Dexin,Jiangxi	Snake-type sampling	0-20	2008.5	Copper	AAS	Wang et al. (2010a)
A21	Suxian, Hunan	Quincunx sampling	0-20		Polymetallic	GFAAS	Song (2014)
A22	Nanjing, Jiangsu	Gridding sampling	0-15	2007.6	Copper	GFAAS	Qin (2009)
A23	Hunan	Multipoint sampling	0-20		Lead-zinc	FAAS	Wu (2007)
A24	Dulong,Yunan	Zigzag sampling	0-20		Polymetallic	AAS	Li et al. (2010a)
A25	Shaoguan, Guangdong	Gridding sampling	0-30		Polymetallic	AAS	Su et al. (2016)

A26	Hunan	Quincunx sampling	5-20		Lead-zinc	GFAAS	Huang (2016)
A27	Hunan	Multipoint sampling	0-20	2005.9	Lead-zinc	ICP-MS	Ji et al. (2008)
A28	Fushun, Liaoning	Multipoint sampling	0-20		Copper	AAS	Shi et al. (2010)
A29	Fushun, Liaoning	Multipoint sampling	0-20		Coal	ICP-OES	Wei et al. (2008)
A30	Fengxian, Shaanxi	Snake-type sampling	0-20		Lead-zinc	FAAS	Chen (2010)
A31	Jiangxi	Snake-type sampling	0-20		Tungsten	ICP-AES	Zhang (2015)
A32	Jiangxi	Random sampling	0-20	2008.10	Tungsten	AAS	Ou (2010)
A33	Jiangxi	Snake-type sampling	0-20		Tungsten	ICP-MS	Chen et al. (2015)
A34	Zhijin, Guizhou	Sampling along the River	0-20		Coal		Li (2012)
A35	Shaoguan,Guangdong	Multipoint sampling	0-20		Polymetallic	ICP-MS	Zheng et al. (2008)
A36	Bayi, Guangxi	Quincunx sampling	0-20	2006.1	Manganese	AAS	Lai et al. (2006)
A37	Chehe, Guangxi	Snake-type sampling	0-20		Polymetallic		Wang et al. (2016)
A38	Daxin, Guangxi	Random sampling	0-20		Manganese	AAS	Li et al. (2010b)
A39	Huanjiang, Guangxi	Multipoint sampling	0-20		Lead-zinc	ICP-MS	Zhai et al. (2008a)
A40	Debao, Guangxi	Crossover interval sampling	0-15	2007.5	Iron	AAS	Wang et al. (2008a)
A41	Guipin, Guangxi	Quincunx sampling	0-20		Manganese	AAS	Chen et al. (2014)
A42	Hechi, Guangxi	Chessboard-type sampling	0-15	2008.11	Lead-zinc	ICP-MS	Xiang et al. (2010)
A43	Guangxi	Multipoint sampling	0-20	2005.10-2006.1	Manganese	AAS	Yang et al. (2007)
A44	Lipu, Guangxi	Gridding sampling	0-20	2004.5-2004.10	Manganese	ICP	Zhang et al. (2006)
A45	Nandan, Guangxi	Multipoint sampling	0-20		Tin	ICP-AES	Liu et al. (2012)
A46	Guizhou	Random sampling	0-20	2010.9	Mercury	GFAAS	Liu et al. (2016a)
A47	Guizhou	Quincunx sampling	0-20		Lead-zinc	AAS	Wu et al. (2016)
A48	Niujiaotang, Guizhou	Snake-type sampling	15-20	2010.12	Lead-zinc	GFAAS	Mao et al. (2014)
A49	Guizhou	Multipoint sampling	0-20		Lead-zinc	ICP-AES	Wu et al. (2009)
A50	Magu, Guizhou		0-20	2014.6	Lead-zinc		Zheng et al. (2015)
A51	Guibei, Guangxi	Multipoint sampling	0-20	2004.5-2005.11	Manganese	ICP	Luo et al. (2007)
A52	Chenzhou, Hunan	Random sampling	0-20	2014.9-2014.11	Manganese	AAS	Pan et al. (2015)
A53	Hanyuan, Sichuan	Quincunx sampling	0-20		Lead-zinc	FAAS	Feng (2007)
A54	Shizhuyuan, Hunan		0-20		Polymetallic	FAAS	Yue (2004)

A55	Huainan, Anhui	Gridding sampling	0-20		Coal	AAS	Guan (2011)
A56	Lengshuijiang, Hunan	Random sampling	10-20	2008.10	Antimony	AAS	She et al. (2010)
A57	Huainan, Anhui	Quincunx sampling	0-20		Coal	ICP-MS	Liu (2015)
A58	Liancheng, Fujian	Snake-type sampling	0-20		Manganese	ICP-MS	Si (2012)
A59	Jiutai, Jilin	Quincunx sampling	0-20		Coal	FAAS	Li (2014a)
A60	Panshi, Jilin	Random sampling	0-20	2013-2014.9	Nickel	GFAAS	Jia (2016)
A61	Xuzhou, Jiangsu	Multipoint sampling	0-20	2006.3-2007.9	Coal		Dong (2008)
A62	Tonglin, Anhui	Multipoint sampling	0-20	2011.4-2011.11	Copper	ICP	Dou et al. (2015)
A63	Panshi, Jilin	Random sampling	0-20	2013.9	Nickel	GFAAS	Jia et al. (2015)
A64	Jilin	Random sampling	0-20	2013.9	Nickel	GFAAS	Liu et al. (2015)
A65	Pinshuo, Shanxi	Multipoint sampling	0-20	2003.7	Coal	GFAAS	Qin (2004)
A66	Lipu, Zhejiang	Snake-type sampling	0-20		Copper	AAS	Long et al. (2003)
A67	Liaoning	Snake-type sampling	0-20		Polymetallic	AAS	Shi (2010)
A68	Tielin, Liaoning	Multipoint sampling	0-20		Coal	AAS	Liu (2011)
A69	Pinshuo, Shanxi	Snake-type sampling	0-20		Coal	AAS	Ge et al. (2008)
A70	Chongqing	Multipoint sampling	0-20		Manganese	AAS	Yu et al. (2016)
A71	Hebei		0-15		Coal	ICP-MS	Fan et al. (2011)
A72	Huludao, Liaoning	Gridding sampling	0-20	2007.5.10	Molybdenum	ICP-AES	Cong et al. (2009)
A73	Huludao, Liaoning	Gridding sampling	0-20	2006.4.10	Molybdenum	ICP-AES	Qu et al. (2008)
A74	Guangxi		0-20	2012.7	Polymetallic	GFAAS	Yu et al. (2015)
A75	Guiyang, Guizhou	Diagonal sampling	0-20	2012.3.21	Coal	AAS	Li (2014b)
A76	Changhua,Hainan	Quincunx sampling	0-20		Lead-zinc	ICP-MS	Lu (2013)
A77	Huludao, Liaoning	Gridding sampling	0-20	2006.4.13	Molybdenum	ICP-OES	Qu (2007)
A78	Tonglin, Anhui	Diagonal sampling	25-40		Copper	FAAS	Shen (2006)
A79	Hanyuan, Sichuan	Diagonal sampling	0-20		Lead-zinc	AAS	Zhao (2012)
A80	Niujiaotang, Guizhou	Crossover interval sampling	0-20	2009.10	Lead-zinc	AAS	Mao et al. (2011)
A81	Yongzhou, Hunan		0-20	2001-2005	Lead-zinc	FAAS	Peng et al. (2007)
A82	Chuxiong, Yunnan	Crossover interval sampling	0-20		Arsenic	AAS	Yi et al. (2011)
A83	Shenmu, Shannxi	Snake-type sampling	0-20	2009.9	Coal	ICP	Shi et al. (2013)
A84	Shaoxing, Zhejiang		0-20		Lead-zinc	AAS	Tian (2006)

A85	Shenmu, Shannxi	Snake-type sampling	0-20	2009.9	Coal	ICP	Shi (2011)
A86	Panzhihua, Sichuan		0-5		Iron	ICP-OES	Xu (2009)
A87	Pinshuo, Shanxi	Gridding sampling	0-30	2013.8	Coal	FAAS	Yu (2015)
A88	Jixi, Heilongjiang	Crossover interval sampling	0-20	2008.7	Graphite		Han et al. (2011)
A89	Tongling, Anhui	Diagonal sampling	25-40		Copper	FAAS	Shen et al. (2005)
A90	Guizhou	Quincunx sampling	0-20	2011.7-2013.8	Lead-zinc	AAS	Jiang et al. (2016)
A91	Sanmeng, Zhejiang		0-20	2006.10	Lead-zinc	ICP-AES	Dai et al. (2013)
A92	Wanzhuang, Beijing		0-20		Gold	ICP	Zhang et al. (2014)
A93	Zhejiang	Sampling along the River	0-15	2014.6	Lead-zinc	GFAAS	Lu et al. (2015a)
A94	Dabaoshan, Guangdong	Snake-type sampling	0-20		Polymetallic	ICP-OES	Zhang et al. (2010)
A95	Hanyuan, Sichuan	Random sampling	0-20	2013.7	Lead-zinc	GFAAS	Zhou et al. (2014)
A96	Lengshuijiang, Hunan	Random sampling	0-20	2011.10	Antimony	AAS	Xue (2013)
A97	Tongling, Anhui	Sampling vertical the River	0-20		Copper	ICP	Yang (2007)
A98	Tongling, Anhui	Multipoint sampling	0-20		Copper	GFAAS	Wang (2014)
A99	Fuzhou, Jiangxi	Multipoint sampling	0-25	2014.7-2015.9	Uranium	ICP-MS	Wang (2016)
A100	Xiangtan, Hunan		0-20	2013.6	Manganese	AAS	Lin (2015)
A101	Huayuan, Hunan	Snake-type sampling	0-20		Lead-zinc	AAS	Zhu (2012)
A102	Xiangtan, Hunan		0-20	2004.3-2004.4	Manganese	AAS	Yan et al. (2006)
A103	Xiangxi, Hunan	Multipoint sampling	0-20		Lead-zinc	ICP-MS	Ji et al. (2009)
A104	Huayuan, Hunan	Quincunx sampling Snake-type sampling	0-30	2010.10	Manganese	ICP-AES	Yang et al. (2012)
A105	Huayuan, Hunan	Quincunx sampling	0-20		Lead-zinc	AAS	Liu et al. (2011)
A106	Xuzhou, Jiangsu	Chessboard-type sampling	0-20		Coal	FAAS	Li et al. (2010c)
A107	Miyun, Beijing	Random sampling	5-20		Gold	ICP-OES	Gao (2012)
A108	Chenzhou, Hunan	Quincunx sampling	5-20	2012	Lead-zinc	GFAAS	Huang (2013)
A109	Daxing, Guangxi	Quincunx sampling	0-20	2013.3	Lead-zinc	ICP	Lv et al. (2013)
A110	Changhua, Hainan	Quincunx sampling	0-20	2011.6-2011.8	Lead-zinc	ICP-MS	Lu (2012)

A111	Meixian, Guangdong	Multipoint sampling	0-20	2010.11-2010.12	Coal	AAS	Liu et al. (2013)
A112	Tiantai, Zhejiang	Random sampling	20-40		Lead-zinc	AAS	Teng et al. (2004)
A113	Xiangtan,Hunan	Gridding sampling	0-45		Manganese	AAS	Liu et al. (2009)
A114	Hunan	Quincunx sampling	0-20		Lead-zinc	GFAAS	Hu et al. (2014b)
A115	Guizhou	Random sampling	0-30		Zinc	GFAAS	Yang et al. (2010)
A116	Youxi, Fujian	Gridding sampling	0-20		Lead-zinc	AAS	Guo et al. (2015)
A117	Aletai, Xinjiang	Random sampling	0-20		Copper-zinc	FAAS	Liu et al. (2016b)
A118	Tongliao, Inner Mongolia	Gridding sampling	0-20	2014.5	Uranium	GFAAS	Haribala et al (2016)
A119	Jiutai, Jilin		0-20		Coal	FAAS	Tang et al. (2012)
A120	Miyun, Beijing	Multipoint sampling	0-20		Gold	ICP-MS	Li et al. (2014a)
A121	Huainan, Anhui	Random sampling	10-30		Coal	ICP-MS	You et al. (2016)
A122	Jiangsu	Random sampling	15-20	2012.5	Polymetallic	ICP-MS	Ma et al. (2016)
A123	Huize, Yunnan	Multipoint sampling	0-20	2012.3	Lead-zinc	ICP-MS	Qi et al. (2016)
A124	Lipu,Guangxi		0-20	2004.5-2004.11	Manganese	ICP-AES	Li et al. (2007)
A125	Gejiu, Yunnan		0-20	2013.4	Tin	FAAS	Li et al. (2014b)
A126	Dayi, Sichuan	Quincunx sampling	0-15		Lead-zinc	GFAAS	Lu et al. (2014)
A127	Baoji, Shannxi		0-20	2012.11-2013.2		ICP-AES	Deng et al. (2016)
A128	Huainan, Anhui		0-20	2010.10	Coal	FAAS	Niu et al. (2015a)
A129	Quzhou, Zhejiang	Snake-type sampling	0-20		Lead-zinc	GFAAS	Yan et al. (2015)
A130	Shaoguan, Guangdong	Multipoint sampling	0-20	2007.9	Polymetallic		Zhuang (2009)
A131	Baotou, Inner Mongolia	Gridding sampling	0-20		Iron	ICP-MS	Pan et al. (2016)
A132	Tongling, Anhui	Quincunx sampling	0-20		Copper	ICP-MS	Ye et al. (2015)
A133	Chenzhou, Hunan	Multipoint sampling	0-20		Lead-zinc	GFAAS	Zhai et al. (2008b)
A134	Huize, Yunnan	Multipoint sampling	0-20	2011.4	Lead-zinc	ICP-AES	Lu et al. (2015b)
A135	Xuzhou, Jiangsu	Snake-type sampling	0-10	2006.3-2007.9	Coal	FAAS	Dong et al. (2012)
A136	Huainan, Anhui		0-20	2010.10	Coal	FAAS	Niu et al. (2015b)
A137	Jiangsu	Gridding sampling	0-15		Copper	AAS	Qin et al. (2012)
A138	Xiangtan, Hunan	Quincunx sampling	0-20		Manganese	AAS	OuYang et al. (2016)
A139	Dexing,Jiangxi	Random sampling	0-20	2014.10	Copper	GFAAS	Jian et al. (2016)
A140	Hunan		0-20	2012.5	Lead-zinc	AAS	Lu et al. (2015c)

A141	Chenzhou, Hunan	Random sampling	0-10		Lead-zinc		Lei et al. (2009)
A142	Guangxi	Snake-type sampling	0-20		Polymetallic	AAS	Luo et al. (2011)
A143	Gejiu, Yunnan	Gridding sampling	0-20		Tin	ICP	Huang et al. (2014)
A144	Dabaoshan, Guangdong	Multipoint sampling	0-10	2009.7	Tin	AAS	Zhuang et al. (2014)
A145	Niujiaotang, Guizhou		0-20	2009.10	Lead-zinc	AAS	Mao et al. (2011)
A146	Ganluoxian, Sichuan	Multipoint sampling	0-20	2006.4-2006.10	Lead-zinc	AAS	Yang et al. (2011b)
A147	Zhaoyuan, Shandong	Gridding sampling	2-20	2007.5	Gold	ICP-OES	Wang et al. (2010b)
A148	Daye, Hubei		0-20		Polymetallic	GFAAS	Li et al. (2016)
A149	Xiangtan, Hunan	Random sampling	0-30		Manganese	FAAS	Wang et al. (2008b)
A150	Dachang, Guangxi		0-20	2006.4	Polymetallic	ICP-MS	Zhang et al. (2008)
A151	Hezhang, Guizhou	Multipoint sampling	0-20	2008	Zinc	GFAAS	Li et al. (2011)
A152	Guangdong	Crossover interval sampling	0-15		Lead-zinc	AAS	Liu et al. (2012)
A153	Daye, Hubei	Random sampling	0-60	2012.9	Copper	AAS	Kang et al. (2014)
A154	Tiantai, Zhejiang	Multipoint sampling	0-20		Lead-zinc	AAS	Cheng et al. (2015)

AAS, Atomic Absorption Spectrophotometer, FAAS, Flame Atomic Absorption Spectrophotometer, GFAAS, Graphite Furnace Atomic Absorption Spectrometry, ICP, Inductively Coupled Plasma, ICP-OES, Inductively Coupled Plasma-Optical Emission Spectroscopy, ICP-AES, Inductively Coupled Plasma-Atomic Emission Spectroscopy, ICP-MS, Inductively Coupled Plasma Mass Spectrometer; Blanks represent values which couldn't be found in the references.

Table 2. Parameters of blood lead model in this study.

	$PbB_{adult,0}$	BKSF (μg /dL per μg /day)	EF_S (days/year)	IR_S (g/day)	AF_S
Pb	1.95	0.4	250	0.1	0.12

Table 3. Basic information of 63 major mining areas.

No	Region	Longitude	Latitude	Provinces	Mineral species
1	Tongling Copper Mining Area	117.700000	30.753333	Anhui	Copper
2	Upper Miyun Reservoir Gold Mining Area	115.933333	40.516667	Beijing	Gold
3	Wanzhuang Gold Mining Area	116.954444	40.227778	Beijing	Gold
4	Chengkou Manganese Mining Area	108.578232	31.833333	Chongqing	Manganese
5	Liancheng Manganese Mining Area	116.537222	25.226389	Fujian	Manganese
6	Youxi Lead-Zinc Mining Area	117.800000	25.833333	Fujian	Lead-Zinc
7	Dabaoshan Mining Area	113.666667	24.500000	Guangdong	Polymetallic
8	Lead-Zinc Mining Area of Bingcun, Meixian County	116.276111	24.362222	Guangdong	Lead-Zinc
9	Debao Mining Area	106.150000	23.016667	Guangxi	Iron
10	Daxin Lead-Zinc Mining Area	106.666667	22.500000	Guangxi	Lead-Zinc
11	Nandan Mining Area	107.016667	24.716667	Guangxi	Polymetallic
12	Daxin Manganese Mining Area	107.207680	22.836471	Guangxi	Manganese
13	Dachang Town Mining Area	107.576944	24.823889	Guangxi	Polymetallic
14	Chehe Mining Area	107.647500	24.856111	Guangxi	Polymetallic
15	Huanjiang Lead-Zinc Mining Area	107.850000	24.733333	Guangxi	Lead-Zinc
16	Hechi Lead-Zinc Mining Area	108.033333	24.700000	Guangxi	Lead-Zinc
17	Bayi Manganese Mining Area	109.287872	23.889605	Guangxi	Manganese
18	Guiping Manganese Mining Area	110.294722	23.430556	Guangxi	Manganese
19	Lipu Manganese Mining Area	110.391667	24.550000	Guangxi	Manganese
20	Lead-Zinc Mining Area in Northwest Guizhou	103.603333	27.783333	Guizhou	Lead-Zinc
21	Magu Town Lead-Zinc Mining Area	104.516667	26.933333	Guizhou	Lead-Zinc
22	Niujiaotang Lead-Zinc Mining Area, Duyun	107.633228	26.216208	Guizhou	Lead-Zinc
23	Changhua Lead-Zinc Mining Area	108.633333	18.883333	Hainan	Lead-Zinc
24	Daye Copper Mining Area	114.943342	30.076930	Hubei	Copper
25	Huahuan Mining Area, Western Hunan	109.183333	27.733333	Hunan	Manganese
26	Huahuan Lead-Zinc Mining Area	109.252333	28.166667	Hunan	Lead-Zinc
27	Fenghuang Lead-Zinc Mining Area	109.425556	27.831111	Hunan	Lead-Zinc
28	Lengshuijiang Antimony Mining Area	111.315833	27.513611	Hunan	Antimony
29	Yongzhou Lead-Zinc Mining Area	111.350000	26.260000	Hunan	Lead-Zinc

30	Shuikoushan Lead-Zinc Mining Area, Hengyang	112.150000	26.266667	Hunan	Lead-Zinc
31	Shizhuyuan Mining Area, Chenzhou	112.216667	24.883333	Hunan	Manganese
32	Zhongnan Antimony Mining Area	112.333333	24.883333	Hunan	Antimony
33	Xiangtan Manganese Mining Area	112.616667	27.666667	Hunan	Manganese
34	Chenzhou Lead-Zinc Mining Area	113.033333	25.800000	Hunan	Lead-Zinc
35	Qingjiang Lead-Zinc Mining Area	113.283333	25.750000	Hunan	Lead-Zinc
36	Hongqiling and Piaohechuan Nickel Mining Area	126.066667	43.266667	Jilin	Nickel
37	Qixiashan Lead-Zinc Mining Area, Nanjing	119.096111	32.082500	Jiangsu	Lead-Zinc
38	Jiuhuashan Copper Mining Area, Nanjing	119.060000	32.046389	Jiangsu	Copper
39	Suzhou Western Mining Area	120.300000	31.200000	Jiangsu	Polymetallic
40	Tungsten Mining Area in South Jiangxi	114.297466	25.400174	Jiangxi	Tungsten
41	Xiangshan Uranium Mining Area	115.866667	27.416667	Jiangxi	Uranium
42	Dexing copper Mining Area	117.000000	28.833333	Jiangxi	Copper
43	Huludao Molybdenum Mining Area	120.852333	40.752333	Liaoning	Molybdenum
44	Dagushan Mining Area	123.064942	41.070875	Liaoning	Iron
45	Fushun Copper Mining Area	124.515438	41.995733	Liaoning	Copper
46	Baotou Aobao Mining Area	109.253333	40.248889	Inner Mongolia	Iron
47	Tongliao Uranium Mining Area	122.566667	43.925000	Inner Mongolia	Uranium
48	Guliku gold placer area, Daxinganling	125.516111	50.859722	Inner Mongolia	Gold
49	Zhaoyuan Gold Mining Area	120.333333	37.416667	Shandong	Gold
50	Yindongliang Lead-Zinc Mining Area, Fengxian County	106.604167	33.876389	Shaanxi	Lead-Zinc
51	Panzhihua Vanadium Titanium Magnetite Mining Area	101.133333	26.083333	Sichuan	Iron
52	Hanyuan Lead-Zinc Mining Area	102.266667	29.083333	Sichuan	Lead-Zinc
53	Chipugou Lead-Zinc Mining Area, Ganluo County	102.460556	28.640000	Sichuan	Lead-Zinc
54	Dayi Lead-Zinc Mining Area	103.333333	30.666667	Sichuan	Lead-Zinc
55	Aletai Mining Area	86.366667	48.266667	Xinjiang	Lead-Zinc
56	Huize Mining Area	103.050000	25.800000	Yunnan	Lead-Zinc
57	Gejiu Mining Area	103.101111	23.106667	Yunnan	Tin
58	Dulong Mining Area	103.866667	22.700000	Yunnan	Polymetallic
59	A Small Lead-Zinc Mining Area in East China	118.666667	29.616667	Zhejiang	Lead-Zinc
60	Quzhou Upper Mining Area	118.790000	29.221667	Zhejiang	Lead-Zinc
61	Lipu Copper Mining Area	119.985833	29.723056	Zhejiang	Copper

62	Tiantai Lead-Zinc-Silver Mining Area	120.690000	28.950556	Zhejiang	Lead-Zinc
63	Shaoxing Lead-Zinc Mining Area	120.777500	30.003889	Zhejiang	Lead-Zinc

Table 4. Lead pollution and emission coefficients of different mining industries (Data from the Handbook of Pollution Coefficients of Pollutant Source Census).

Mineral type	Scale (t/day)	Pollution coefficient (g/t)	Emission coefficient (g/t)
Tungsten	≥1000t/day	0.103	0.0083
	500-1000t/day	0.11	0.013
	<500t/day	0.106	0.021
Lead-zinc	≥3000t/day	0.7	0.134
	600-3000t/day	0.668	0.2
	<600t/day	0.654	0.262
Antimony	≥3000t/day	0.014	0.002
	600-3000t/day	0.018	0.0035
	<600t/day	0.016	0.0048
Tin	≥3000t/day	0.069	0.01
	600-3000t/day	11.567	0.013
	<600t/day	13.445	0.022
Copper	≥3000t/day	0.015	0.002
	600-3000t/day	0.0012	0.0026
	<600t/day	0.0016	0.004
Gold	≥3000t/day	0.0042	0.0003
	600-3000t/day	0.0048	0.0026
	<600t/day	0.0048	0.004

Table 5. Data extracted from the literature.

Reference	Province	Year	Type	Mean (mg/kg)	No. of sampling
Song et al. (2012)	Yunnan	2011	Tin	573.61	85
Yuan et al. (2015)	Hunan	2015	Antimony	178	18
Huang et al. (2014)	Yunnan	2014	Tin	1157.58	203
Tang et al. (2016)	Guangxi	2016	Manganese	29.93	18
Su et al. (2014)	Guangdong	2013	Polymetallic	450.96	152
Zou et al. (2008)	Guangdong	2007	Polymetallic	843.7	58
Zhou et al. (2004)	Guangdong	2004	Polymetallic	205.39	24
Xu et al. (2008)	Guangdong	2007	Polymetallic	179.93	21
Huang et al. (2009)	Guangdong	2008	Polymetallic	110.91	30
Yang et al. (2011a)	Liaoning	2009	Iron	111.06	11
Hu et al. (2014a)	Inner Mongolia	2014	Gold	28.3	30
Sun et al. (2013)	Hubei	2012	Copper	122.18	6
Lu et al. (2014)	Sichuan	2014	Lead-Zinc	7168.51	10
	Sichuan	2014	Lead-Zinc	750.69	17
Mi et al. (2016)	Yunnan	2016	Tin	293.17	
Chen et al. (2010)	Guizhou	2010	Hydrargyrum	6.16	4
Sun et al. (2012)	Hunan	2012	Lead-Zinc	1595.75	62
Chen et al. (2007)	Jiangxi	2007	Copper	51	919
Wang et al. (2010a)	Jiangxi	2010	Copper	80.75	16
Song. (2014)	Hunan	2014	Non-ferrous	179.6	118
	Hunan	2014	Non-ferrous	180.1	127
	Hunan	2014	Non-ferrous	3390	28
Qin. (2009)	Jiangsu	2011	Copper	53.8	155
	Jiangsu	2011	Lead-Zinc	701	
Wu. (2007)	Hunan	2007	Lead-Zinc	250.841	24
Su et al. (2016)	Guangdong	2015	Polymetallic	1035.42	
	Guangdong	2015	Polymetallic	812.36	
	Guangdong	2015	Lead-Zinc	747.23	
Huang. (2016)	Hunan	2015	Lead-Zinc	1872.05	

	Hunan	2015	Lead-Zinc	1209.3	
	Hunan	2015	Lead-Zinc	305.27	
	Hunan	2015	Lead-Zinc	583.75	
	Hunan	2015	Lead-Zinc	426	
	Hunan	2015	Lead-Zinc	649.11	
	Hunan	2015	Lead-Zinc	878.06	83
Ji et al. (2009)	Hunan	2007	Lead-Zinc	875	14
Shi et al. (2010)	Liaoning	2010	Copper	207	
	Liaoning	2010	Copper	142	
Chen. (2010)	Shaanxi	2009	Lead-Zinc	637.84	8
Zhang. (2015)	Jiangxi	2015	Tungsten	113.2	17
	Jiangxi	2015	Tungsten	61.4	15
	Jiangxi	2015	Tungsten	55.1	17
Ou. (2010)	Jiangxi	2010	Tungsten	25.95	26
Chen et al. (2015)	Jiangxi	2015	Tungsten	330	5
	Jiangxi	2015	Tungsten	313	5
Zheng et al. (2008)	Guangdong	2007	Polymetallic	193	52
Lai et al. (2006)	Guangxi	2006	Manganese	191.84	7
Wang et al. (2016)	Guangxi	2015	Non-ferrous	98.52	11
Li et al. (2010b)	Guangxi	2008	Manganese	72.54	9
Zhai et al. (2008a)	Guangxi	2008	Lead-Zinc	798	
	Guangxi	2008	Lead-Zinc	544	
	Guangxi	2008	Lead-Zinc	650	
	Guangxi	2008	Lead-Zinc	508	
Wang et al. (2008a)	Guangxi	2007	Iron	74.63	3
	Guangxi	2007	Iron	62.9	3
	Guangxi	2007	Iron	75.66	3
	Guangxi	2007	Iron	83.53	3
	Guangxi	2007	Copper	65.13	3
	Guangxi	2007	Copper	58.71	3
	Guangxi	2007	Manganese	52.8	3
Chen et al. (2014)	Guangxi	2014	Manganese	89.25	4~5

	Guangxi	2014	Manganese	88.19	4~5
	Guangxi	2014	Manganese	63.24	4~5
	Guangxi	2014	Manganese	58.69	4~5
Xiang et al. (2010)	Guangxi	2010	Lead-Zinc	485	70
	Guangxi	2010	Lead-Zinc	444	
Yang et al. (2007)	Guangxi	2006	Manganese	178.9	9
	Guangxi	2006	Manganese	202.17	9
	Guangxi	2006	Manganese	240.8	9
Zhang et al. (2006)	Guangxi	2005	Manganese	35.5	
	Guangxi	2005	Manganese	60.2	
	Guangxi	2005	Manganese	25.7	
	Guangxi	2005	Manganese	17.9	
	Guangxi	2005	Manganese	59.1	
	Guangxi	2005	Manganese	66.2	
	Guangxi	2005	Manganese	26.8	
	Guangxi	2005	Manganese	20.9	
Liu. (2012)	Guangxi	2012	Tin	151.262	144
Liu. (2016a)	Guizhou	2016	Hydrargyrum	10.215	
Wu et al. (2016)	Guizhou	2016	Lead-Zinc	1411.67	27
Mao et al. (2014)	Guizhou	2014	Lead-Zinc	323	6
Wu et al. (2009)	Guizhou	2008	Lead-Zinc	1335.74	18
Zheng et al. (2015)	Guizhou	2015	Lead-Zinc	161.25	6
Luo et al. (2007)	Guangxi	2007	Manganese	25.395	8
Pan et al. (2015)	Hunan	2015	Manganese	82.3	3
	Hunan	2015	Manganese	134.2	3
	Hunan	2015	Manganese	378.4	3
	Hunan	2015	Manganese	145.3	3
	Hunan	2015	Manganese	187.9	3
	Hunan	2015	Manganese	579.6	3
	Hunan	2015	Manganese	112.4	3
	Hunan	2015	Manganese	123.2	3
	Hunan	2015	Manganese	456.5	3

Feng. (2007)	Sichuan	2007	Lead-Zinc	240.332	21
She et al. (2010)	Hunan	2010	Antimony	45.4	9
Si. (2012)	Fujian	2012	Manganese	171	167
Jia. (2016)	Jilin	2013	Nickel	25.71	52
Dou et al. (2015)	Anhui	2015	Copper	91.36	147
Jia et al. (2015)	Jilin	2014	Nickel	26.04	40
Liu et al. (2015)	Jilin	2014	Nickel	23.91	27
Long et al. (2003)	Zhejiang	2002	Copper	444.95	3
Shi. (2010)	Liaoning	2010	Copper	164.33	
	Liaoning	2010	Lead-Zinc	2948.67	
	Liaoning	2010	Molybdenum	138.33	
Yu et al. (2016)	Chongqing	2016	Manganese	114.28	16
Cong et al. (2009)	Liaoning	2008	Molybdenum	78.89	80
Qu et al. (2008)	Liaoning	2007	Molybdenum	81.6	20
Yu et al. (2015)	Guangxi	2015	Non-ferrous	1503	27
Lu. (2013)	Hainan	2013	Lead-Zinc	7148.8	20
	Hainan	2013	Lead-Zinc	2055.33	15
Qu. (2007)	Liaoning	2007	Molybdenum	447.98	20
	Liaoning	2007	Molybdenum	398.29	20
	Liaoning	2007	Molybdenum	334.22	20
	Liaoning	2007	Molybdenum	298.67	20
	Liaoning	2007	Molybdenum	78.89	20
Shen. (2006)	Anhui	2006	Copper	57.94	
	Anhui	2006	Copper	52.8	
	Anhui	2006	Copper	73.2	
	Anhui	2006	Copper	91.3	
Zhao. (2012)	Sichuan	2006	Lead-Zinc	4281.07	24
Peng et al. (2007)	Hunan	2006	Lead-Zinc	6884.1	
Tian. (2006)	Zhejiang	2006	Lead-Zinc	189	3
Xu. (2009)	Sichuan	2009	Iron	32	58
Shen et al. (2005)	Anhui	2005	Copper	56.79	
	Anhui	2005	Copper	55.31	

Jiang et al. (2016)	Guizhou	2011	Lead-Zinc	1081.21	3
	Guizhou	2013	Lead-Zinc	772.05	3
Dai et al. (2013)	Zhejiang	2012	Lead-Zinc	3947.285	3
Zhang et al. (2014)	Beijing	2014	Gold	1720	4
	Beijing	2014	Gold	522.63	4
	Beijing	2014	Gold	613.75	4
	Beijing	2014	Gold	202.23	4
	Beijing	2014	Gold	193.83	4
Lu et al. (2015a)	Zhejiang	2015	Lead-Zinc	275	11
	Zhejiang	2015	Lead-Zinc	49.8	11
	Zhejiang	2015	Lead-Zinc	34.8	11
Zhang et al. (2010)	Guangdong	2009	Polymetallic	430.51	
	Guangdong	2009	Polymetallic	1245.47	
Zhou. (2014)	Sichuan	2014	Lead-Zinc	90	2
Xue. (2013)	Hunan	2013	Antimony	100.8	3
	Hunan	2013	Antimony	75.58	3
	Hunan	2013	Antimony	47	3
	Hunan	2013	Antimony	46.69	3
	Hunan	2013	Antimony	35.95	3
Yang. (2007)	Anhui	2007	Copper	73.76	68
Wang. (2014)	Anhui	2014	Non-ferrous	134.75	17
Lin. (2015)	Hunan	2015	Manganese	68.913	
Zhu. (2012)	Hunan	2012	Lead-Zinc	2168.2	18
	Hunan	2012	Lead-Zinc	3269.3	16
	Hunan	2012	Lead-Zinc	482.6	10
	Hunan	2012	Lead-Zinc	2316.9	16
	Hunan	2012	Lead-Zinc	2938.5	12
	Hunan	2012	Lead-Zinc	373.8	10
Yan et al. (2006)	Hunan	2006	Manganese	922.3	3
	Hunan	2006	Manganese	143.5	3
	Hunan	2006	Manganese	524.7	3
	Hunan	2006	Manganese	188.9	3

Ji et al. (2008)	Hunan	2009	Lead-Zinc	99.4	5
	Hunan	2009	Lead-Zinc	2806.3	19
	Hunan	2009	Lead-Zinc	3226	7
Yang et al. (2012)	Hunan	2011	Manganese	511	50
	Hunan	2011	Lead-Zinc	768	58
Liu et al. (2011)	Hunan	2011	Lead-Zinc	1734	
Gao. (2012)	Beijing	2012	Iron	26.3	17
	Beijing	2012	Gold	37.5	20
Huang. (2013)	Hunan	2013	Lead-Zinc	2559.04	14
	Hunan	2013	Lead-Zinc	790.96	31
	Hunan	2013	Lead-Zinc	629.34	12
	Hunan	2013	Lead-Zinc	363.37	20
	Hunan	2013	Lead-Zinc	607.78	17
Lv et al. (2013)	Guangxi	2013	Lead-Zinc	395.29	14
	Guangxi	2013	Lead-Zinc	412.48	11
	Guangxi	2013	Lead-Zinc	89.2	11
Lu. (2012)	Hainan	2012	Lead-Zinc	2055.33	
Teng et al. (2004)	Zhejiang	2002	Lead-Zinc	875.59	8
Liu et al. (2009)	Hunan	2008	Manganese	1808.62	27
Hu et al. (2014b)	Hunan	2013	Lead-Zinc	671	141
Guo et al. (2015)	Fujian	2014	Lead-Zinc	1301.74	19
	Fujian	2014	Lead-Zinc	209.33	17
	Fujian	2014	Lead-Zinc	903.12	21
	Fujian	2014	Lead-Zinc	1866.32	18
Liu et al. (2016b)	Xinjiang	2016	Copper-Zinc	731.5	
	Xinjiang	2016	Copper-Zinc	1162.5	
	Xinjiang	2016	Copper-Zinc	311.5	
	Xinjiang	2016	Copper-Zinc	591	
	Xinjiang	2016	Copper-Zinc	604.5	
	Xinjiang	2016	Copper-Zinc	464	
	Xinjiang	2016	Lead-Zinc	863.5	
	Xinjiang	2016	Lead-Zinc	369.5	

Haribala et al. (2016)	Inner Mongolia	2016	Uranium	34.9	40
Li et al. (2014a)	Beijing	2013	Gold	109	34
Ma et al. (2016)	Jiangsu	2016	Polymetallic	157.3	50
	Jiangsu	2016	Polymetallic	262.2	43
Qi et al. (2016)	Yunnan	2016	Lead-Zinc	3518.4	90
Li et al. (2007)	Guangxi	2007	Manganese	951.6	
Li et al. (2014b)	Yunnan	2014	Tin	893.36	19
Yan et al. (2015)	Zhejiang	2015	Lead-Zinc	542.02	47
Zhuang. (2009)	Guangdong	2008	Polymetallic	278	32
	Guangdong	2008	Polymetallic	190	28
Pan et al. (2016)	Inner Mongolia	2015	Iron	49.49	100
	Inner Mongolia	2015	Iron	33.73	100
Ye et al. (2015)	Anhui	2014	Copper	93.22	10
	Anhui	2014	Copper	184.81	
Lu et al. (2015b)	Yunnan	2014	Lead-Zinc	218.6	396
Qin et al. (2012)	Jiangsu	2012	Copper	53.8	155
OuYang et al. (2016)	Hunan	2016	Manganese	169.2	
	Hunan	2016	Manganese	161.6	
	Hunan	2016	Manganese	145.9	
	Hunan	2016	Manganese	125.3	
	Hunan	2016	Manganese	79.4	
	Hunan	2016	Manganese	136.2	
Jian et al. (2016)	Jiangxi	2016	Copper	9.36	8
Lu et al. (2015c)	Hunan	2015	Lead-Zinc	92.1	150
Lei et al. (2009)	Hunan	2009	Lead-Zinc	1029.8	
	Hunan	2009	Lead-Zinc	1247.2	
Huang et al. (2014)	Yunnan	2014	Tin	95.42	203
	Yunnan	2014	Tin	1157.58	41
	Yunnan	2014	Tin	254.92	31
Zhuang et al. (2014)	Guangdong	2014	Tin	386	122
Mao et al. (2011)	Guizhou	2011	Lead-Zinc	215.9	8
	Guizhou	2011	Lead-Zinc	386.95	9

	Guizhou	2011	Lead-Zinc	94.19	8
	Guizhou	2011	Lead-Zinc	77.52	9
	Guizhou	2011	Lead-Zinc	135.1	9
	Guizhou	2011	Lead-Zinc	110.7	8
	Guizhou	2011	Lead-Zinc	153.6	9
	Guizhou	2011	Lead-Zinc	127.9	9
Yang et al. (2011b)	Sichuan	2011	Lead-Zinc	4095	18
	Sichuan	2011	Lead-Zinc	2238	12
Wang et al. (2010b)	Shandong	2008	Gold	134.41	36
Zhang et al. (2008)	Guangxi	2009	Non-ferrous	1085	23
Li et al. (2011)	Guizhou	2011	Lead-Zinc	337	12
Kang et al. (2014)	Hubei	2014	Copper	137.06	
Cheng et al. (2005)	Zhejiang	2005	Lead-Zinc	996.61	8

Table 6. Data analysis results (unit: mg/kg).

Minimum	Maximum	Mean	Median	Standard Deviation
1.6	7168.51	542.08	188.45	874.3

Table 7. Data of different regions (unit: mg/kg).

	10th	50th	90th	Mean	Standard Deviation
East	50.98	134.75	977.91	431.03	750.69
South	47.61	259.4	7848.38	3000.7	2887.17
Central	21.24	187.4	1833.99	643.617	1029.67
North	26.22	124.7	549.97	324.19	602.4
Southwest	59.31	293.17	3749.04	1094.55	1751.76

Table 8. Data of different years (unit: mg/kg).

	10th	50th	90th	Mean	Standard Deviation
2002	488.01	660.27	832.53	660.27	215.32
2003	-	-	-	39.27	-
2004	-	-	-	205.39	-
2005	20.9	55.31	66.2	129.182	274.83
2006	75.01	239.95	1170.5	587.341	886.34
2007	53.98	83.53	764.56	252.07	273.974
2008	82.09	393	1281.97	542.43	523.86
2009	100.57	1057.4	3184.03	1544.05	1800.54
2010	7.18	37.8	325.5	194.58	564.19
2011	89.19	361.98	1885.2	744.14	1001.54
2012	44.02	482.6	3136.98	1313.98	1323.9
2013	34.93	379.33	917.4	512.27	658.26
2014	60.06	205.78	1594.52	726.64	1320.05
2015	50.3	182.95	925.27	389.99	430.3
2016	31.421	215.7	1072.8	489.88	728

Table 9. Data of different mining areas (unit: mg/kg).

	**10th**	**50th**	**90th**	**Mean**	**Standard Deviation**
Tungsten	8.2	24.7	113.2	60.49	88.72
Lead-Zinc	130.06	781.51	7162.6	2016.14	2582.81
Antimony	41.62	47	131.68	75.63	46.69
Manganese	31.04	145.3	768.86	294.55	327.06
Copper	52.9	73.48	180.53	102.56	87.58
Gold	27.9	134.41	540.85	207.55	203.7
Iron	33.21	68.77	91.79	65.38	24.95
Tin	140.09	386	1157.58	551.43	394.73

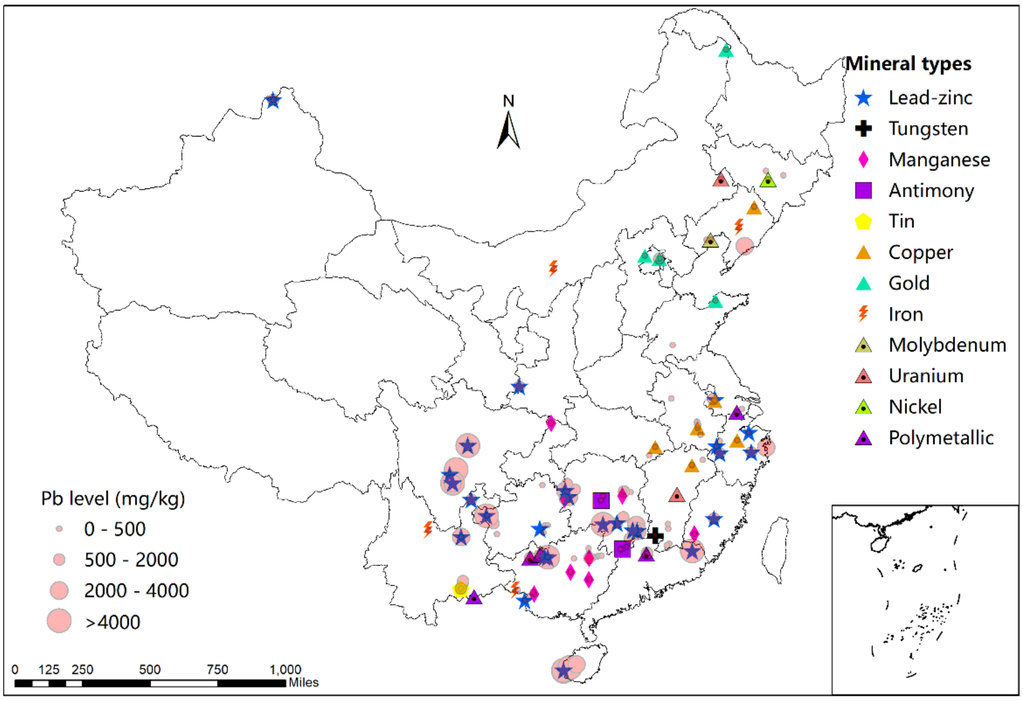

Figure 1. Pollution situation around different mining areas.

References

1. Cheng, L.C., Min, L., Chang Y.H., 2005. Effect of combined pollution by heavy metals on soil enzymatic activities in areas polluted by tailings from Pb-Zn-Ag mine. Journal of Environmental Sciences 17(4), 637-640.
2. Chen, C.H., Ni, S.J., He, B.B., Zhang, C.J., 2007. Heavy Metals Contamination of Soils of in Dexing Mine, Jiangxi Province, China. Earth and environment 35(02), 134-141. (in Chinese with English abstract).
3. Cong, X., Yuan, X., Qu, J., Zhang, H.W., 2009. The Analysis and Assessment on the Pollution Condition of Heavy Metals in the Soil in the Farmland around the Molybdenum Ore Areas. Environmental Monitoring in China 25(01), 7-51. (in Chinese with English abstract).
4. Chen, X.P., Zhang, C.H., 2010. The indicatory potential of Marchantia polymorpha L.for evaluation the heavy metal contamination in Muyouchang Mercury Mine. Environmental Pollution and Control 32(10), 24-28. (in Chinese with English abstract).
5. Chen GD., 2010. Study on the Heavy Metal Pollution of Yindongliang Lead-zinc Mine Area in Fengxian. Xi'an University of Science and Technology. (in Chinese with English abstract).
6. Chen, C.Q., Deng, H., Huang, F.F., 2014. Concentration and Speciation Analysis of Heavy Metals in Soils in Guiping Manganese Mine, Guangxi, China.Journal of Guangxi Normal University:Natural Science Edtion 32(04), 108-114. (in Chinese with English abstract).
7. Chen, M., Yang, T., Xu, H., Cai, Z.P., Zhao, L., 2015. Distribution characteristics and ecological risk assessment of heavy metals Cd and Pb in soils around a tungsten mine of Gannan. Environment Chemistry 34(12), 2257-2262. (in Chinese with English abstract).
8. Dong JH., 2008. Distribution of Heavy Metals in Reclamation Soils and Their Accumulation in Crops. China University of Mining and Technology. (in Chinese with English abstract)
9. Dong, J., Yu, M., Bian, Z., 2012. The Safety Study of Heavy Metal Pollution in Wheat Planted in Reclaimed Soil of Mining Areas in Xuzhou, China. Environ. Earth Sci 66, 673-682.
10. Dai, D.L., Huang, T., Zhang, J.H., 2013. Bioaugmentation with indigenous metal-resistant bacteria to enhance metal mobility of MeiKeng, a Pb /Zn tailing. Journal of Biology 30(02), 51-55. (in Chinese with English abstract)
11. Dou, Z.Y., Cheng, J.H., Zhou, P., Xu, D.C., Sun, Q.Y., 2015. Ecological risk assessment based on total and available content of heavy metals in farmland soil of Tongling mining area. Environmental Pollution & Control 37(11), 6-10. (in Chinese with English abstract).
12. Deng, W., Li, X., An, Z., et al., 2016. The occurrence and sources of heavy metal contamination in peri-urban and smelting contaminated sites in Baoji, China[J]. Environmental Monitoring and Assessment 188(4), 251.
13. Finley, B.L., Scott, P.K., Mayhall, D.A., 1994. Development of a standard soil-to-skin adherence probability density function for use in Monte Carlo analyses of dermal exposure. Risk Anal. 14, 555-569.
14. Feng WJ., 2007. The study of heavy metal forms and bioavailability around Tangjia zink-leak mine in Hanyuan. Chengdu University of Technology. (in Chinese with English abstract).
15. Fan, J.S., Meng, Z.Q., Li, Y.H., Hun, L.Y., Niu, H.Y., 2011. Discussion on Heavy Metal Potential Ecological Risk of the Coal Gangue Dump of one Mine. Industrial Safety and Environmental Protection 37(06), 11-12. (in Chinese with English abstract).
16. Ge, Y.Y., Cui, X., Bai, Z.K., 2008. Evaluation on Potential Ecological Risk of Heavy Metal Pollution in Reclaimed Soil of Opencast——Taking Pingshuo Opencast Mine as an Example. Journal of Shanxi Agricultural University (Natural Science Edition) 01, 85-88. (in Chinese with English abstract).
17. Guan Y., 2011. Study on the Distributional Characteristics of Heavy Metals in the Environmental Remediation Soil of Huainan Mining Areas. Anhui University of Science and Technology. (in Chinese with English abstract).
18. Gao YX., 2012. Heavy metal pollution and risk assessment in soil of metal mining area in the upper reaches of Miyun reservoir, Beijing. Capital Normal University. (in Chinese with English abstract).
19. Guo, S.H., Hou, X.L., Qiu, H.Y., Liu, A.Q., Ma, X.Q., Wang, Y.S., 2015. Speciation characteristics and risk assessment of heavy metals in soils from different functional zones of the lead-zinc mine. Geological Bulletin of China 34(11), 2047-2053. (in Chinese with English abstract).
20. HC, 2004. Federal Contaminated Site Risk Assessment in Canada-Part II: Health Canada Toxicological Reference Values (TRVs) and Chemical-Specific Factors, Ottawa, Canada.
21. Huang, S.H., Tian, T., Zou, X.J., Zhou, X.Y., Palaninaiaker Senthilkumar., Qiu, R.L., 2009. Bioavailability Assessment of Heavy Metals in the Vicinity of Dabaoshan Mine. Acta Scientiarum Naturalium Universitatis Sunyatseni 48(04), 125-129. (in Chinese with English abstract).

22. Han, X.B., Wang, X.F., Cai, T.J., 2011. Characteristics and evaluation of heavy metal pollution of graphite tailings reservoir and its surrounding soil. Journal of Engineering of Heilo ng jiang Unive rsity 2(02), 58-62. (in Chinese with English abstract).
23. Huang SH., 2013. Assessment of heavy metal pollution and potential ecological hazards in soils of typical non-ferrous metal mining areas [a]. China Academy of Environmental Sciences. Proceedings of 2013 China Academy of Environmental Sciences Annual Meeting (Volume IV) [C]. China Academy of Environmental Sciences: China Academy of Environmental Sciences 7. (in Chinese with English abstract).
24. Hu, Y.L., Wang, C.Y., Wang, H.Q., Zeng, D.H., 2014a. Characteristics of soil heavy metals concentrations in the tailings of Guliku placer gold mine in the Greater Khingan Mountains. Chinese Journal of Ecology 33(10), 2796-2802. (in Chinese with English abstract).
25. Hu, M., Wu, J.Q., Peng, P.Q., Gan, G.J., Zhou, H., Liao, B.H., 2014b. Assessment model of heavy metal pollution for arable soils and a case study in a mining area. Acta Scientiae Circumstantiae 34(02), 423-430. (in Chinese with English abstract).
26. Huang, Y., Cai, B.X., Wang, Y., Li, H.X., Chai, J.L., 2014. The contribution of the mining activities on the heavy metal accumulation in soils of the Gejiu tin deposit, Yunnan Province. Geological Bulletin of China 33(8), 1167-1174. (in Chinese with English abstract).
27. Haribala., Hu, B., Wang, C., et al., 2016. Assessment of radioactive materials and heavy metals in the surface soil around uranium mining area of Tongliao, China. Ecotoxicology and Environmental Safety 130, 185-192.
28. Huang SH., 2016. Study on pollution characteristics and effectiveness of Heavy Metals in Soil of discarded around a Lead and Zinc Mining Area. Technological Development of enterprise 35(04), 5-7. (in Chinese with English abstract).
29. Ji, Y.F., Li, Y.H., Sun, H.F., Yang, L.S., Wang, W.Y., 2008. Translocation and Accumulation of Heavy Metals in Soil-Paddy System at Fenghuang Lead-Zinc Deposit Area. Journal of Agro-Environment Science 27(06), 2143-2150. (in Chinese with English abstract).
30. Ji, Y.F., Li, Y.H., Yang, L.S., Sun, H.F., Wang, W.Y., 2009. Spatial distribution and ecological significance of heavy metals in typical soil profiles in the Fenghuang Lead-Zinc mining area, Western Hunan Province. Acta Scientiae Circumstantiae 29(05), 1094-1102. (in Chinese with English abstract).
31. Jia, L., Liu, J.H., Zhang, L., et al., 2015. Characteristics of Heavy Metal Pollution in Farmland Soil of the Two Nickel Mines of Hongqiling and Piaohechuan, Jilin Province. Journal of Jilin Agricultural University 37(3),338-345. (in Chinese with English abstract).
32. Jian, M.F., Yang, Y.P., Yu, H.P., Ye, J.M., Jin, C., 2016. Heavy Metal Enrichment and Bioaccumulation Characteristics of Boehmeria nivea, a Dominant Species of Plant Growing in Dexing Mining Area of Jiangxi Province. Journal of Ecology and Rural Environment 32(03), 486-491. (in Chinese with English abstract).
33. Jia, L., 2016. Pollution Characteristics and Migration of Heavy Metals in Soil-Corn System Located in Nickel Mining Area. Jilin University. (in Chinese with English abstract).
34. Jiang, H., He, B., Luo, Q., Zhang, Y.Z., Zhao, Y.S., Gan, G.D., Shi, K.J., LV, T., 2016. Correlation Analysis on Heavy Metal Pollution and Advantages of Plant from Wasteland of Artisanal Lead-zinc Melter by Indigenous Method. Hubei Agricultural Sciences 55(15), 3875-3879. (in Chinese with English abstract).
35. Kang, W., Bao, J.G., Zheng, J., Zou, T., Min, J.H., Yang, Y.Q., 2014. Analysis on heavy metal enrichment ability of woody plants at ancient copper mine site in Tonglushan of Hubei Province. Journal of Plant Resources and Environment 23(01), 78-84. (in Chinese with English abstract).
36. LaGoy, P.K., 1987. Estimated soil ingestion rates for use in risk assessment. Risk Anal. 7, 355-359.
37. Long, J., Huang, C.Y., Teng, Y., Yao, G.Y., 2003. Effects of Heavy Metal Pollution on Microbial Indicators in Soil of a Mining. Journal of Agro-Environment Science 01, 60-63. (in Chinese with English abstract).
38. Lai, Y.P., Li, M.S., Yang, S.X., Chen, C.Q., Li, Y., 2006. Heavy metal concentrations in soils and main agronomic crops in restored Bayi manganese mine, Guangxi. Mineral Resources and Geology 06, 651-655. (in Chinese with English abstract).
39. Liu, H.X., Wang, X.Y., Hang, B.B., 2004. Soil Pollution Evaluation with Heavy Metals in Baodian Coal Field of Yanzhou Mineral Industry Group. Energy Environmental Protection 02, 56-58. (in Chinese with English abstract).
40. Luo, Y.P., Wu, X.F., Li, M.S., Yi, R.Z., 2007. Investigation of main plant species and assessment of soil heavy metal pollutions in manganese mine wastelands in north Guangxi. Ecology and Environment 04, 1149-1153. (in Chinese with English abstract).

41. Li, M.S., Luo, Y.P., Su, Z.Y., 2007. Heavy metal concentrations in soils and plant accumulation in a restored manganese mineland in Guangxi, South China. Environmental Pollution 147(1), 0-175.
42. Liu, X., Yan, W.D., Xiang, W.H., 2009 Heavy-metal Contents and Absorption Characteristics of Plants in the Soil of Xiangtan Manganese Mine Wasteland. Journal of Central South University of Forestry & Technology 29(02), 25-29. (in Chinese with English abstract).
43. Lei, M., Zhang, Y., Khan, Sardar., Qin, P.F., Liao, B.H., 2009. Pollution, fractionation, and mobility of Pb, Cd, Cu, and Zn in garden and paddy soils from a Pb/Zn mining area. Environmental monitoring and assessment 168, 215-222.
44. Li, F.W., Bei, R.T., Wu, M., 2010a. Study on Cadmium & Zinc Pollution of Soils and Plants in Dulong Mine. Modern Agricultural Science and Technology 22, 263-265. (in Chinese with English abstract).
45. Li, J.C., Yi, R.Z., Luo, Y.P., Lu, Y.L., Zhang, L.D., 2010b. Assessment of Heavy Metal Contamination of Soils in Daxin Manganese Mine Guangxi. Environmental Science & Technology 33(07), 183-185. (in Chinese with English abstract).
46. Li, B.J., Yu, F.Z., Ji, Y.Z., 2010c. Calculation of shield tunnel central plane coordinates. Science of Surveying and Mapping 35(06), 166-169. (in Chinese with English abstract).
47. Li, Z.G., Feng, X.F., Bi, X.Y., Sun, G.Y., Cui, L.F., Wang, J.X., Liu, T.Z., 2011. Present situation of soil heavy metals contamination in an artisanal zinc smelting zone of Guizhou, China. Chinese Journal of Ecology 30(05), 897-901.
48. Luo, H., Fan, Z.L., Mo, L.Y., Zhang, X.L., 2011. Accumulation of heavy metals in plants grown in abandoned mines in Guangxi. Journal of Southern Agriculture 42(07), 765-767. (in Chinese with English abstract).
49. Liu, Z., 2011. Pollution Characteristics of Heavy Metal and Environment Risk Assessment in Soil of Tiefa Mining in Liaoning Province. Anhui Normal University. (in Chinese with English abstract).
50. Liu, C., Zou, D.S., Zhu, J.W., 2011. Investigation of Contaminated Soil and Plants by Heavy Metals in Xiangxi Pb-Zn Mining Area. Journal of Anhui Agricultural Sciences 39(35), 21743-21746. (in Chinese with English abstract).
51. Lu, S.F., 2012.Soil heavy metal elements content characteristics and pollution evaluation in Changhua lead-zinc mine tailings wasteland of Hainan. Hainan Normal University. (in Chinese with English abstract).
52. Liu, Y., 2012. Analysis and Assessment on the Heavy Metal Content and Pollution of Farmland Soil in A Mining Field of Guangxi. Nanning Normal University. (in Chinese with English abstract).
53. Liu, H.N., Yang, Q.H., Yang, H.S., Li, J.Q., Liu, D.L., 2012. Characteristics of absorption and accumulation of heavy metals for three dominant plants in Pb-Zn mine tailings eastern Guangdong. Guihaia 32(06), 743-749. (in Chinese with English abstract).
54. Li, J., 2012. Study on pollution characters and migration rules of heavy metals on high-sulphur coal gangue abandoned land. China University of Mining and Technology. (in Chinese with English abstract).
55. Lu, B.B., 2013. Study on soil heavy metal elements content and Enrichment Characteristics of Dominant Plants in Changhua lead-zinc mine wasteland of Hainan. Hainan Normal University. (in Chinese with English abstract).
56. Liu, D.L., Yang, Q.H., 2013. Appraisal for Environmental Quality of Soil Polluted by Heavy Metals in Wasteland of Coalmine Tailing in Mingshan of Guangdong. Hubei Agricultural Sciences 52(18), 4351-4354. (in Chinese with English abstract).
57. Lv, J.J., Zhang, X.Y., Wu, Y.F., Zeng, B.H., 2013. Characteristics and Assessment of Heavy Metal Pollution in the Arable Soil from the Village of Daxin Lead Zinc Mining Area. Journal of Guangxi Teachers Education University:Natural Science Edition 30(04), 51-54. (in Chinese with English abstract).
58. Li, H.J., 2014a. Evaluation of Heavy Metal Contaminated Soil and the Effect of Leguminous Plants Remediation in Typical Mining Wasteland in Jilin Province. Jilin University. (in Chinese with English abstract).
59. Li, B.J., 2014b. Evaluation of Heavy Metal Contaminated Soil and the Effect of Dominant Plants Remediation in Mining Wasteland in Guizhou Mountain District. Southwest University. (in Chinese with English abstract).
60. Li, Q., Ji, H., Qin, F., et al., 2014a. Sources and the distribution of heavy metals in the particle size of soil polluted by gold mining upstream of Miyun Reservoir, Beijing: implications for assessing the potential risks. Environmental Monitoring and Assessment 186(10), 6605-6626.
61. Li, Y., Wang, H., Wang, H., et al., 2014b. Heavy metal pollution in vegetables grown in the vicinity of a multi-metal mining area in Gejiu, China: total concentrations, speciation analysis, and health risk. Environmental Science and Pollution Research 21(21), 12569-12582.

62. Lu, L., Yang, J.Y., Tian, LY., Yang, J., Tang, Y., 2014. Status Quo and Evaluation of Heavy Metal Pollution of Vegetables and Soils in Dayi Lead-Zinc Mining Area, China. Journal of Ecology and Rural Environment 30(03), 374-380. (in Chinese with English abstract).
63. Lin, Y., 2015. The Research on Ecological Restoration and Its Ecological Benefits in XiangTan Manganese Mining Wasteland. Central South University of Forestry and Technology. (in Chinese with English abstract).
64. Liu, Y.J., 2015. Research on Spatial Distribution and Contamination Evaluation of Heavy Metal Pb, Cd, Hg in Huainan Mining Area Based on. Hefei University of Technology. (in Chinese with English abstract).
65. Lu, Y.F., Li, Y.F., Miao, X.Q., Zhou, T., Duan, L.L., Shang, Z.C., 2015a. Heavy Metal Pollution of Soil and Plants in Areas of Micro-Sized Lead-Zinc Mine and Risk Assessment. Journal of Ecology and Rural Environment 31(04), 566-571. (in Chinese with English abstract).
66. Lu, S.J., Teng, Y.G., Wang, Y.Y., et al., 2015b. Research on the ecological risk of heavy metals in the soil around a Pb-Zn mine in the Huize County, China. Chinese journal of geochemistry 34(4).
67. Lu, S., Wang, Y., Teng, Y., et al., 2015c. Heavy metal pollution and ecological risk assessment of the paddy soils near a zinc-lead mining area in Hunan. Environmental Monitoring and Assessment 187(10), 627.
68. Liu, J.H., Jia, L., Zhang, L., Xu, C.L., Xie, Z.L., 2015. Accumulation and distribution of heavy metals in maize and its relationship with total heavy metals in soil. Environmental Protection and Circular Economy 35(05), 48-52. (in Chinese with English abstract).
69. Liu, R.X., 2016a. Heavy metal pollution and biotransfer characteristics of soil crops in mercury mining area of Southeast Guizhou. Gansu Science and Technology 32(09), 31-35. (in Chinese with English abstract).
70. Liu Z, Hamuti A, Abdulla H, et al., 2016b. Accumulation of metallic elements by native species thriving in two mine tailings in Aletai, China. Environmental Earth Sciences 75(9), 781.
71. Li, M., Mohamed I, Raleve D., et al., 2016. Field evaluation of intensive compost application on Cd fractionation and phytoavailability in a mining-contaminated soil. Environmental Geochemistry and Health 38(5), 1193-1201.
72. MADEP, 2002. Technical Update: Calculation of Enhanced Soil Ingestion Rate. Boston. Off. Res. Stand.
73. Mao, H.L., Long, C.M., Chen, G.C., et al., 2011. Absorption and Accumulation Characteristics of Heavy Metals by Plants in Lead-Zinc Mining Area. Environmental Science & Technology 34(12), 114-118.
74. Mao, H.L., Long, C.M., Yang, Y., Zou, H.T., 2014. Speciation and Distribution Characteristics of Heavy Metals in Soil of Guizhou Niujiaotang Lead-Zinc Mining Area. Environmental Science & Technology 37(S2), 15-19. (in Chinese with English abstract).
75. Ma, Z.W., Chen, K., Li, Z.Y., Bi, J., Huang, L., 2016. Heavy metals in soils and road dusts in the mining areas of Western Suzhou, China: a preliminary identification of contaminated sites. Journal of Soils and Sediments 16(1), 204-214.
76. Mi, Y.H., Lei, M., Li, Q.W., Chen, L., Du, L.J., Deng, X.X., Yang, X.K., Zhang, W.B., 2016. Phytoremediation and Health Risk Assessment of Heavy Metals Pollution in Mining Area Farmland of Southern Yunnan Province. Ecology and Environmental Sciences 25(5), 864-871. (in Chinese with English abstract).
77. Niu, S.P., Gao, L.M., Zhao, J.J., 2015a. Risk Analysis of Metals in Soil from a Restored Coal Mining Area[J]. Bulletin of Environmental Contamination and Toxicology 95(2), 183-187.
78. Niu, S.P., Gao, L.M., Zhao, J.J., 2015b. Distribution and Risk Assessment of Heavy Metals in the Xinzhuangzi Reclamation Soil from the Huainan Coal Mining Area, China, Human and Ecological Risk Assessment: An International Journal 21,4, 900-912
79. Ou, L., 2010. Study on Phytoremediation and Physiological Characteristic of Cadmium Contaminated Soil of Gannan Tungsten Mine. Nanchang University. (in Chinese with English abstract).
80. OuYang, L.N., Wu, X.F., Li, Y., Feng, C.L., Chen, Y.H., 2016. Growth and heavy metal accumulation of Paulownia fortunei and Koelreuteria bipinnata in an ecological restoration site of the manganese-ore tailing. China Environmental Science 36(03), 908-916. (in Chinese with English abstract).
81. Peng, H.B., Liu, Y.G., Li, A.Y., 2007. Forms of heavy metals in lead zinc ore tailings and its potential ecological risk. Journal of Hunan Agricultural University (Natural Sciences) 03, 345-347. (in Chinese with English abstract).
82. Pan, Q., Pan, F., 2015. Status and assessment of heavy metal pollution in the soil of metallurgical cities in Hunan Province. Jiangsu Agricultural Sciences 43(10), 405-410. (in Chinese with English abstract)

83. Pan, Y.X., Li, H.T., 2016. Investigating Heavy Metal Pollution in Mining Brownfield and Its Policy Implications: A Case Study of the Bayan Obo Rare Earth Mine, Inner Mongolia, China. Environmental Management 57, 879-893
84. Qin, J.M., 2004. Analysis and Evaluation of Reclaimed Soil's Environmental Quality of Mine Area—Taking Pingshuo Opencast Mine as an Example. Shanxi Agricultural University. (in Chinese with English abstract)
85. Qu, J., 2007. The Analysis and Assessment on the Pollution Condition of Heavy Metals in the Soil and Farmland of the Molybdenum Mine. Northeast Normal University. (in Chinese with English abstract)
86. Qu, J., Wang, H.Y., Yuan, X., Cong, Q., Zhang, H.W., 2008. Heavy metal content and the ecological risk warning assessment of the vegetable land-soil near the tailing-areas of molybdenum ore. Journal of Safety and Environment 02, 76-79. (in Chinese with English abstract)
87. Qin, C., 2009. Contamination of Heavy Metals in Soils and vegetables in the Typical and Smelting Circumjacent Districts. Nanjing Agricultural University. (in Chinese with English abstract).
88. Qin, C., Luo, C., Chen, Y., et al., 2012. Spatial-Based Assessment of Metal Contamination in Agricultural Soils Near an Abandoned Copper Mine of Eastern China. Bulletin of Environmental Contamination and Toxicology 89(1), 113-118.
89. Qi, J.Y., Zhang, H.L., Li, X.P., et al., 2016. Concentrations, spatial distribution, and risk assessment of soil heavy metals in a Zn-Pb mine district in southern China. Environmental Monitoring and Assessment 188(7).
90. Smith, R.L., 1994. Use of Monte Carlo simulation for human exposure assessment at a superfund site. Risk Anal. 14, 433-439.
91. Shen, Z.J., Wang, Y.B., Wang, G.L., Yan, M., Li, Z., Liu, D.Y., 2005. Heavy metals pollution of Paeonia ostii land at copper-tailings reservoir of Tongling city:A preliminary study. Chinese Journal of Applied Ecology 04, 673-677. (in Chinese with English abstract).
92. Shen, Z.J., 2006. Study on heavy metals pollution of Paeonia ostia planting land in the copper-tailings wasterland in Tongling. Anhui Normal University. (in Chinese with English abstract).
93. Shi, P., 2010. Research on Soil Heavy Metal Pollution and Phytoremediation in Representative Nonferrous Metal Mine Area of Liaoning Province. Northeastern University. (in Chinese with English abstract).
94. Shi, P., Wei, Z.Y., Jiang, L., Wang, E.D., 2010. Reserch on Tolerance of Plant to Heavy Metal is Wastelands of Fushun Hongtoushan Copper Mine. Metal Mine 02, 155-158. (in Chinese with English abstract).
95. She, W., Jie, Y.C., Xing, H.C., Huang, M., Kang, W.L., Lu, Y.W., Wang, D., 2010. Uptake and Accumulation of Heavy Metal by Ramie（Boehmeria nivea） Growing on Antimony Mining Area in Lengshuijiang City of Hunan Province. Journal of Agro-Environment Science 29(01), 91-96. (in Chinese with English abstract).
96. Shi, Z.F., 2011. Study on the Soil Physiochemical Properties and Vegetation in Shenmu Mining Area. Northwest A&F University. (in Chinese with English abstract).
97. Si, H.J., 2012.Study on the Ecological Risk Assessment and Precaution of Heavy Metal in the Farming Soil of Manganese Mining Area Based on GIS Technology. Fujian Agriculture and Forestry University. (in Chinese with English abstract).
98. Song, Y.H., Zhong, Z.Y., Li, H.M., Wang, H.B., 2012. Heavy-metal pollution in-situ in farming soil-crop system in multi-metal mining area—a case study of Zhadian Town, Gejiu City, Yunnan. Journal of Safety and Environment 12(01), 138-146. (in Chinese with English abstract).
99. Sun, Y., Shu, F., Sun, W.L., Hao, W., Li, L., 2012. Heavy Metal Contamination in Paddy Soils around a Pb-Zn Mining and Smelting Area and Its Relation with Soil Physicochemical Properties. Acta Scientiarum Naturalium Universitatis Pekinensis 48(01), 139-146. (in Chinese with English abstract).
100. Sun, Q.B., Yin, C.Q., Deng, J.F., Zhang, D.F., 2013. Characteristics of soil-vegetable pollution of heavy metals and health risk assessment in Daye mining area. Environmental Chemistry 32(04), 671-677. (in Chinese with English abstract).
101. Shi, Z.F., Wang, L., 2013. Contents of Soil Heavy Metals and Evaluation on the Potential Pollution Risk in Shenmu Mining Area. Journal of Agro-Environment Science 32(06), 1150-1158. (in Chinese with English abstract).
102. Song, D.P., 2014. Heavy Metal Pollution and Resident Heavy Risk Assessment around Metal Mine, a Case Study in Suxian of Hunan. Nanjing Agricultural University.
103. Su, W.J., Fang, S., 2014. Analysis and evaluation of heavy metal pollution along the river of Dabaoshan mine area. Hebei Journal of Industrial Science and Technology 31(03), 199-203. (in Chinese with English abstract).

104. Su, Y.M., Chen, Z.L., Lei, G.J., Fang, X.H., 2016. Vertical Pollution Characteristic and Ecological Risk Assessment of Heavy Metal of Soil Profiles in Polymetallic Ore Mine [J]. Ecology and Environmental Sciences 25(1), 130-134. (in Chinese with English abstract).
105. Teng, Y., Huang, C.Y., Luo, Y.M., Long, J., Yao, K.Y., 2004. Microbial Activities and Functional Diversity of Community in Soils Pollution with Pb-Zn-Ag Mine Tailings. Acta Pedologica Sinica 01, 113-119. (in Chinese with English abstract).
106. Tian, Z.J., 2006. Enrichment of Heavy Metals in Particulate Organic Matter in a Lead/Zinc Mining Contaminated Soil. Zhejiang University. (in Chinese with English abstract).
107. Tang, J., Li, Z.Y., Li, H.J., et al., 2012. The repair effect and accumulation feature of five heavy metals on three forage grass species in the coalmine wasteland. World Automation Congress. IEEE.
108. Tian, L., Wu, H.F., Deng, H.J., Cheng, D.M., Bai, X.W., Li, J.H., 2013. Ecological Risks of Heavy Metal Contamination in Soils Around the Coal Gangue Dump in the Coal Mining Area by Toxicity Characteristic Leaching Procedure. Guizhou Agricultural Sciences 41(01), 123-127. (in Chinese with English abstract).
109. Tang, W.J., Deng, H., Li, H., Zhou, Y.C., 2016. Contamination Characteristics of Soil Heavy Metals in Bayi Mn Mine on Restored Area (Tea Garden). Hubei Agricultural Sciences 55(04), 855-858. (in Chinese with English abstract).
110. United States Environmental Protection Agency (USEPA), 2003. Recommendations of the Technical Review Workgroup for Lead for an Approach to Assessing Risks Associated with Adult Exposures to Lead in Soil. USEPA, Washington, DC
111. Wu, C.H., 2007. Study on the complex pollution of soil by heavy metals near the representative lead-zinc smelt mills. Hunan University of Science and Technology. (in Chinese with English abstract).
112. Wei, Z.Y, Lu, L., Wang, Q.B., 2008. Research on the Heavy Metal Contamination of Funshun West Coal Wastes Pile and its Adjacent Soil. Chinese Journal of Soil Science 04, 946-949. (in Chinese with English abstract).
113. Wang, S.J., Li, Z.W., Liao, Q.J., Tao, Z.F., 2008a. A study on contamination status of the Cd and Pb in the mines soil of GuangXi. Ecological Science 01, 50-54. (in Chinese with English abstract).
114. Wang, X., Liu, Y., Zeng, G., et al., 2008b. Pedological characteristics of Mn mine tailings and metal accumulation by native plants. Chemosphere 72(9), 0-1266.
115. Wang, Z., Liu, S.Q., Chen, X.M., Lin, C.Y., 2008c. Estimates of the exposed dermal surface area of Chinese in view of human health risk assessment. J. Saf. Environ. 8, 152-156 (in Chinese with English abstract).
116. Wang, Z., Duan, X., Liu, P., Nie, J., Huang, N., Zhang, J.L., 2009. Human exposure factors of Chinese people in environmental health risk assessment. Res. Environ. Sci. 22, 1164-1170 (in Chinese with English abstract).
117. Wu, Z.Q., Gu, S.Y., Li, H.Y., Wu, H., 2009. Research on the Heavy Metal Contamination and Bioavailability of Contaminated Soils in Pb-Zn Mining Areas of Northwestern Guizhou Province. Safety and Enviro nmental Engineering 16(03), 1-5. (in Chinese with English abstract).
118. Wang, Z.L., Xie, X.H., Wang, H.P., Zhen, C.L., Liu JS., 2010a. Combined pollution character of heavy metals in soils around a typical copper tailing. Ecology and Environmental Sciences 19(01), 113-117. (in Chinese with English abstract).
119. Wang, Q., Dai, J., Yu, Y., et al., 2010b. Efficiencies of different microbial parameters as indicator to assess slight metal pollutions in a farm field near a gold mining area. Environmental Monitoring & Assessment 161(1-4), 495-508.
120. Wang, Y.Q., 2014. Pollution Assessment and Source Apportionment of Heavy Metals in Soils around XinQiao Mining Area in Tongling, Anhui Province. Hefei University of Technology. (in Chinese with English abstract).
121. Wang, C., Wang, Z., Jiang, X.S., Zhu, M.J., Yao, J., 2016. Heavy Metal Pollution and the Ecological Risk Assessment of Surface Soil in Chehe Mine Area of Guangxi Province. Sichuan Environment 35(02), 92-97. (in Chinese with English abstract).
122. Wang, Y.T., 2016. The Study on the Heavy Metal Cd Content in Paddy Soil and Surface Water of the Xiangshan Uranium Mine Area. East China University of Technology. (in Chinese with English abstract).
123. Wu, H.F., Tian, L., Deng, H.J., Zhang, X.Y., Wang, Y.L., 2016. Heavy Metal Pollution Assessment of Soils of a Pb-Zn Mine in Guizhou. Journal of Liupanshui Normal University 28(04), 18-22. (in Chinese with English abstract).
124. Xu, C., Xia, B.C., He, S.M., Qin, J.Q., Li, H.S., Lin, X.F., 2008. Characteristics of Cd, Zn, Pb, Cu Content of Paddy Soils in the Lower Stream of Dabaoshan Area, Guangdong. Acta Scientiarum Naturalium Universitatis Sunyatseni 03, 122-127. (in Chinese with English abstract).

125. Xu, Z.Q., 2009. Geochemical Characteristics of Heavy Metals in Different Media in Panzhihua V-Ti-Magnetite Zone. Chengdu University of Technology. (in Chinese with English abstract).
126. Xiang, M., Zhang, GP., Li, L., Wei, X.F., Li, H.X., 2010. The Characteristics of Heavy Metals in Soil around the Hechi Antimony-Lead Smelter, Guangxi, China. Earth and Environment 38(04), 495-500. (in Chinese with English abstract).
127. Xiang, M.D., Yu, Y.J., Li, Q., Sun, P., Deng, F., 2012. Exposure factors of residents around Taihu Lake drinking water source site. Res. Environ. Sci. 25, 179-185 (in Chinese with English abstract).
128. Xue, L., 2013. Research on characteristic of heavy metal accumulation and antimony tolerant mechanism of plant in antimony mine area. Chinese Academy of Forestry. (in Chinese with English abstract).
129. Yue, Q.L., 2004. Study on Heavy Metal of Soils and Plants in mining in Hu Nan. Northwest A&F University. (in Chinese with English abstract).
130. Yan, W.D., Tian, D.L., 2006. Relationship between Enzyme Activities and Heavy Metal Contents in Soils of Deserted Land in Xiangtan Manganese Mine. Journal of central south forestry college 03, 1-4. (in Chinese with English abstract).
131. Yang, S.X., Li, M.S., Lai, Y.P., Luo, Y.P., Chen, C.Q., Li, F., 2007. Dominant Plants and Their Heavy Metal Concentrations in Manganese Mine Wastelands, Guangxi. Journal of Guangxi Normal University:Natural Science Edition 01, 108-112. (in Chinese with English abstract).
132. Yang, X.F., 2007. A Current Research on Heavy Metal Pollution to the Soil and Rice in the Farmland in Tongling Mining District. Hefei University of Technology. (in Chinese with English abstract).
133. Yang, Y., Li, S., Bi, X., et al., 2010. Lead, Zn, and Cd in slags, stream sediments, and soils in an abandoned Zn smelting region, southwest of China, and Pb and S isotopes as source tracers. Journal of Soils and Sediments 10(8), 1527-1539.
134. Yi, F., Wang, H.B., Gao, J.P., Pan, Y.H., Jiao, P., 2011. Uptake and accumulation of heavy metals in dominant plants growing in an arsenic-contaminated area. Journal of safety and Environment 11(04), 14-22. (in Chinese with English abstract).
135. Yang, W., Shen, A.L., Li, X., Fen, J.L., 2011a. Distribution Characteristic of Heavy Metal Chemical Form and Its Influencing Factors around the Dagushan Mine District. Journal of Shenyang Jianzhu University (Natural Science) 27(01), 130-134. (in Chinese with English abstract).
136. Yang, G., Shen, F., Zhong, G.J., Xie, L.P., Wang, Y.J., Wu, J., 2011b. Concentration and health risk of heavy metals in crops and soils in a zinc-lead mining area in southwest mountainous regions. Acta Scientiae Circumstantiae 31(09), 2014-2021. (in Chinese with English abstract).
137. Yang, S.X., Yuan, Z.Z., Li, C.Y., Long, H., Tang, W.J., 2012. Heavy Metal Contamination and Bioavailability in Huayuan Manganese and Lead/Zinc Mineland, Xiangxi. Environmental Science 33(05), 1718-1724. (in Chinese with English abstract).
138. Yuan, C., Zhang, H.Z., Chi, T., Yu, F., Song, J., Wu, L.H., 2015. Heavy Metal and Metalloid Pollution of Soils and Plants in Typical Antimony Mining Area of Central-south China. Soils 47(05), 960-964. (in Chinese with English abstract).
139. Ye, W.L., Chen, Z., Xu, X.Y., 2015. Heavy Metal Contents and Enrichment Characteristics of Dominant Plants in Copper Mine Tailings in Tongling of Anhui Province. Environmental Science & Technology 38(05), 11-14. (in Chinese with English abstract).
140. Yan, W.B., Qaisar Mahmood., Peng, D.L., Fu, W.J., Chen, T., Wang, Y., Li, S., Chen, J.R., Liu, D., 2015. The spatial distribution pattern of heavy metals and risk assessment of moso bamboo forest soil around lead–zinc mine in Southeastern China. Soil and Tillage Research 153, 120-130.
141. Yu, Y.Y., Huang, Y.F., Song, B., Xu, T., Lu, S.F., Yuan, Z.N., 2015. Investigation and health risk assessment of heavy metal content in soil and agricultural products around the mining area of Nandan county. Environment Chemistry 34(11), 2133-2135. (in Chinese with English abstract).
142. Yu, G., 2015. Analyze on spatial distribution characteristics and content of soil heavy metal in PingShou mining area. China University of Geosciences, Beijing. (in Chinese with English abstract).
143. You, M., Huang, Y., Lu, J., et al., 2016. Fractionation characterizations and environmental implications of heavy metal in soil from coal mine in Huainan, China. Environmental Earth Sciences 75(1), 78.
144. Yu, S.S., Wang, X.M., Zhu, M.Z., Gao, L.Z., Xia, F.Y., 2016. Analysis and Evaluation on Environmental Characteristics of Soil in Manganese Ore Mining Area. Resource Development and Market 32(10), 1188-1191. (in Chinese with English abstract).
145. Zhou, J.M., Dang, Z., Si, T.Y., Liu, C.Q., 2004. Distribution and Characteristics of Heavy Metals Contaminations in Soils from Dabaoshan Mine Area. Journal of Agro-Environment Science 06, 1172-1176. (in Chinese with English abstract).

146. Zou, X.J., Qiu, R.L., Zhou, X.Y., Zheng, W.H., 2008. Heavy metal contamination and health risk assessment in Dabao Mountain, China. Acta. Scien. Circum. 28, 1406-1412. (in Chinese with English abstract).
147. Zhang, X.H., Liu, J., Huang, H.T., Zhu, Y.N., 2006. Bioaccumulation of Heavy Metals by Dominant Plants Growing in Lipu Manganese Mine, Guangxi, China. Earth and Environment 01, 13-18. (in Chinese with English abstract).
148. Zheng, J.J., Jiang, X., Zhang, X.J., 2008. Pollution Assessment of Heavy Metals in Soil around Dabaoshan Polymetallic Ore Deposit. Environmental Science & Technology 11, 137-139 (in Chinese with English abstract).
149. Zhang, X.Y., Tang, L.S., Zhang, G., Wu, H.D., 2008. Heavy Metal Contamination in a Typical Mining Town of a Minority and Mountain Area, South China. Bulletin of environmental contamination and toxicology 82, 31-38.
150. Zhai, L.M., Chen, T.B., Liao, X.Y., Yan, X.L., Wang, L.X., Xie, H., 2008a. Pollution of agricultural soils resulting from a tailing spill at a Pb-Zn mine: A case study in Huanjiang, Guangxi Province.Acta Scientiae Circumstantiae 06, 1206-1211. (in Chinese with English abstract).
151. Zhai, L.M., Liao, X.Y., Chen, T.B., et al., 2008b. Regional assessment of cadmium pollution in agricultural lands and the potential health risk related to intensive mining activities: A case study in Chenzhou City, China. Journal of Environmental Sciences 20(6), 696-703.
152. Zhuang, P., 2009. Heavy metal contamination in soils and food crops around Dabaoshan mine in Guangdong, China: implication for human health[J]. Scientia Horticulturae 6(31), 707-715.
153. Zhang, W.P., Xu, C., Xia, B.C., Wu, H.N., Liao, Y.L., Tang, H.T., 2010. Species Distribution and Biological Effectiveness of Heavy Metals in Contaminated Soils of Mine Tailing Area. Hunan Agricultural Sciences 01, 54-56. (in Chinese with English abstract).
154. Zhu, J.W., 2012. The Research on the Eco-Remediation of Heavy Metals Contaminated Soils in Huayuan Pb/Zn Mine Area of Xiangxi. Hunan Agricultural University. (in Chinese with English abstract).
155. Zhao, H.Y., 2012. Phytoremediation of co-cropping different plants on mine soil contaminated by lead and zinc. Sichuan Agricultural University. (in Chinese with English abstract).
156. Zhou, S.S., 2014. Environmental Geochemical Characteristics of Lead-Zinc Mine in Hanyuan SiChuan. Chengdu University of Technology. (in Chinese with English abstract).
157. Zhang, A.X., Nie, Y.N., Ji, H.B., Feng, J.G., Qin, F., 2014. Vertical Distribution, Morphological Characteristics of Heavy Metals in Soils of Wanzhuang Gold Mine Field. Environmental Science & Technology 37(S2), 1-8. (in Chinese with English abstract)
158. Zhuang, P., Lu, H., Li, Z., Zou, B., McBride MB., 2014. Multiple Exposure and Effects Assessment of Heavy Metals in the Population near Mining Area in South China. PLoS ONE 9(4), 94484.
159. Zhang, J., 2015. Distribution of heavy metals and their chemical speciation of tungsten mine area soil in south of Jiangxi Province. Jiangxi University of Science and Technology. (in Chinese with English abstract).
160. Zheng, L.P., Wang, G.Q., Lin, Y.S., et al., 2015. Evaluation of toxicity effects of heavy metal contaminated soils on earthworm (Eisenia foetida) in a mining area of Guizhou Province. Asian Journal of Ecotoxicology 10(2), 258-265 (in Chinese with English abstract).
